# Visualizing the pH in Escherichia coli Colonies via the Sensor Protein mCherryEA Allows High-Throughput Screening of Mutant Libraries

**DOI:** 10.1128/msystems.00219-22

**Published:** 2022-04-18

**Authors:** Fabian Stefan Franz Hartmann, Tamara Weiß, Jing Shen, Dóra Smahajcsik, Simonas Savickas, Gerd Michael Seibold

**Affiliations:** a Department of Biotechnology and Biomedicine, Section for Synthetic Biology, Technical University of Denmark, Kongens Lyngby, Denmark; b Department of Biotechnology and Biomedicine, Section for Protein Science and Biotherapeutics, Technical University of Denmark, Kongens Lyngby, Denmark; c Biognosys AG, Schlieren, Switzerland; University of California San Diego

**Keywords:** mCherryEA, ratiometric biosensor, robotic, high-throughput screening, pH homeostasis, *Escherichia coli*

## Abstract

Cytoplasmic pH in bacteria is tightly regulated by diverse active mechanisms and interconnected regulatory processes. Many processes and regulators underlying pH homeostasis have been identified via phenotypic screening of strain libraries for nongrowth at low or high pH values. Direct screens with respect to changes of the internal pH in mutant strain collections are limited by laborious methods, which include fluorescent dyes and radioactive probes. Genetically encoded biosensors equip single organisms or strain libraries with an internal sensor molecule during the generation of the strain. Here, we used the pH-sensitive mCherry variant mCherryEA as a ratiometric pH biosensor. We visualized the internal pH of Escherichia coli colonies on agar plates by the use of a GelDoc imaging system. Combining this imaging technology with robot-assisted colony picking and spotting allowed us to screen and select mutants with altered internal pH values from a small transposon mutagenesis-derived E. coli library. Identification of the transposon (Tn) insertion sites in strains with altered internal pH levels revealed that the transposon was inserted into *trkH* (encoding a transmembrane protein of the potassium uptake system) or *rssB* (encoding the adaptor protein RssB, which mediates the proteolytic degradation of the general stress response regulator RpoS), two genes known to be associated with pH homeostasis and pH stress adaptation. This successful screening approach demonstrates that the pH sensor-based analysis of arrayed colonies on agar plates is a sensitive approach for the rapid identification of genes involved in pH homeostasis or pH stress adaptation in E. coli.

**IMPORTANCE** Phenotypic screening of strain libraries on agar plates has become a versatile tool to understand gene functions and to optimize biotechnological platform organisms. Screening is supported by genetically encoded biosensors that allow to easily measure intracellular processes. For this purpose, transcription factor-based biosensors have emerged as the sensor type of choice. Here, the target stimulus initiates the activation of a response gene (e.g., a fluorescent protein), followed by transcription, translation, and maturation. Due to this mechanistic principle, biosensor readouts are delayed and cannot report the actual intracellular state of the cell in real time. To capture rapid intracellular processes adequately, fluorescent reporter proteins are extensively applied. However, these sensor types have not previously been used for phenotypic screenings. To take advantage of their properties, we established here an imaging method that allows application of a rapid ratiometric sensor protein for assessing the internal pH of colonies in a high-throughput manner.

## INTRODUCTION

Genetically encoded sensors targeting intracellular metabolites have become a versatile tool for physiological studies in diverse organisms (1–3). These sensors have been successfully applied in bacteria for screening optimized production strains and activity of/or resistance against antimicrobial compounds, as well as for assessing physiological states and metabolic fluxes (4–8). Commonly, the following two different types of genetically encoded sensors are used: transcription-factor-based biosensors (TFBs) and fluorescent reporter proteins (FRPs). TFBs are the most extensively developed and applied biosensors due to their simplicity to engineer. The basic design generally relies on transcription factors, which natively react to effectors (activator or repressor). Upon interaction, the TF-effector complex targets or releases a cognate promoter sequence to transduce a response through activation or repression of the respective downstream reporter gene, such as a fluorescent protein coding gene. The dynamics of TFBs applicable to monitoring changes of target product concentrations in real-time are limited, as the sensor signal depends on transcription, translation, maturation and degradation of the fluorescent protein. This, however, provides the advantage that the sensor signal is stable even when a sensor strain is exposed to varying external conditions, making these type of sensors suited for high-throughput screening of strains via, e.g., fluorescence-activated cell sorting (FACS) (5, 9). In contrast, FRPs respond in real-time to alterations of internal target parameters such as pH or metabolites such as ATP or NADPH (10–13). Here, a previously produced sensor protein undergoes analyte-dependent conformational changes accompanied by a change of the fluorescence properties (14, 15). Consequently, FRPs were successfully applied for real-time monitoring of internal metabolite levels or oxidation states upon externally applied perturbations (10, 14–18). Measurement of the internal parameters of individual microbial strains of mutant collections via FRPs could benefit from the fact that actual values can be measured, rather than events which occurred in the past. Furthermore, rapid sensor dynamics would allow the scientist to perform a sensor calibration and validation of sensor properties at the level of the actual screening. However, applying FRPs for FACS-based high-throughput screening of microbial strains is challenging due to varying external conditions. To take advantage of the properties of FRPs, the screening method needs to allow maintenance of constant conditions while conducting the sensor analysis, such as phenotypic screening on agar plates.

Many bacterial regulatory mechanisms have been identified via phenotypic screening of strain libraries with respect to its growth patterns under different conditions. Screening of Escherichia coli and other microorganisms for growth versus nongrowth at low or high pH values revealed many of the processes and control mechanisms underlying pH homeostasis (19–24). To achieve pH homeostasis, E. coli possesses regulatory networks for acid and alkaline conditions, which trigger expression of distinct sets of genes (25, 26). For response to acid conditions, E. coli activates systems for consumption of intracellular protons via deamination and decarboxylation of amino acids, formation of neutralizing ammonia from glutamine, and extrusion of protons via the F_1_Fo-ATPase (27–29). Moreover, potassium uptake and accumulation were shown to be essential for the maintenance of internal pH in E. coli. Under acidic conditions, a neutral pH in the cytoplasm can only be maintained if sufficient potassium is available, accumulated via one of three potassium uptake systems (11, 30). Upon exposure to alkaline pH, E. coli expresses genes for cation proton antiporters, which import protons in exchange for sodium and/or potassium ions (31, 32). Following the identification of a mutant strain possessing a pH-dependent growth phenotype, the cytoplasmic pH of the isolated mutant is measured via fluorescent dyes (e.g., BCECF and SNARF), radioactive probes (33, 34), or genetically encoded biosensors based on fluorescent proteins (15, 35) or firefly luciferases (36). For this purpose, different ratiometric pH-responsive FRPs have been developed, such as pHluorin and pHred, both of which possess a pK_a_ of 6.9 but have different intrinsic fluorescence properties (15, 37, 38). Recently, the mCherry variant mCherryEA was shown to be an effective ratiometric red fluorescent protein pH biosensor possessing a pK_a_ of 7.3 (35). This is close to the range of internal pH values reported for E. coli (7.4 to 7.9) (39), making this sensor protein well suited for applications in E. coli.

Here, we successfully visualized ratiometric sensor signals from the genetically encoded pH sensor mCherryEA in E. coli colonies cultivated on agar plates by using an imaging system equipped with filters for fluorescence detection. Combining this imaging technology with robot-assisted colony picking and spotting allowed us to screen and select mutants with altered internal pH levels from a small transposon mutagenesis-derived E. coli library. We show here that a sensor analysis with the pH sensor mCherryEA of colonies on agar plates is a sensitive approach for the rapid identification and characterization of genes involved in pH homeostasis or pH stress adaption in E. coli. The approach established here can easily be adapted for other strain backgrounds or genetically encoded FRPs targeting another product or internal parameter and thus enables novel studies in microbial systems biology.

## RESULTS AND DISCUSSION

### The plasmid-encoded sensor protein mCherryEA allows real-time monitoring of internal pH in E. coli.

**FIG 1:**
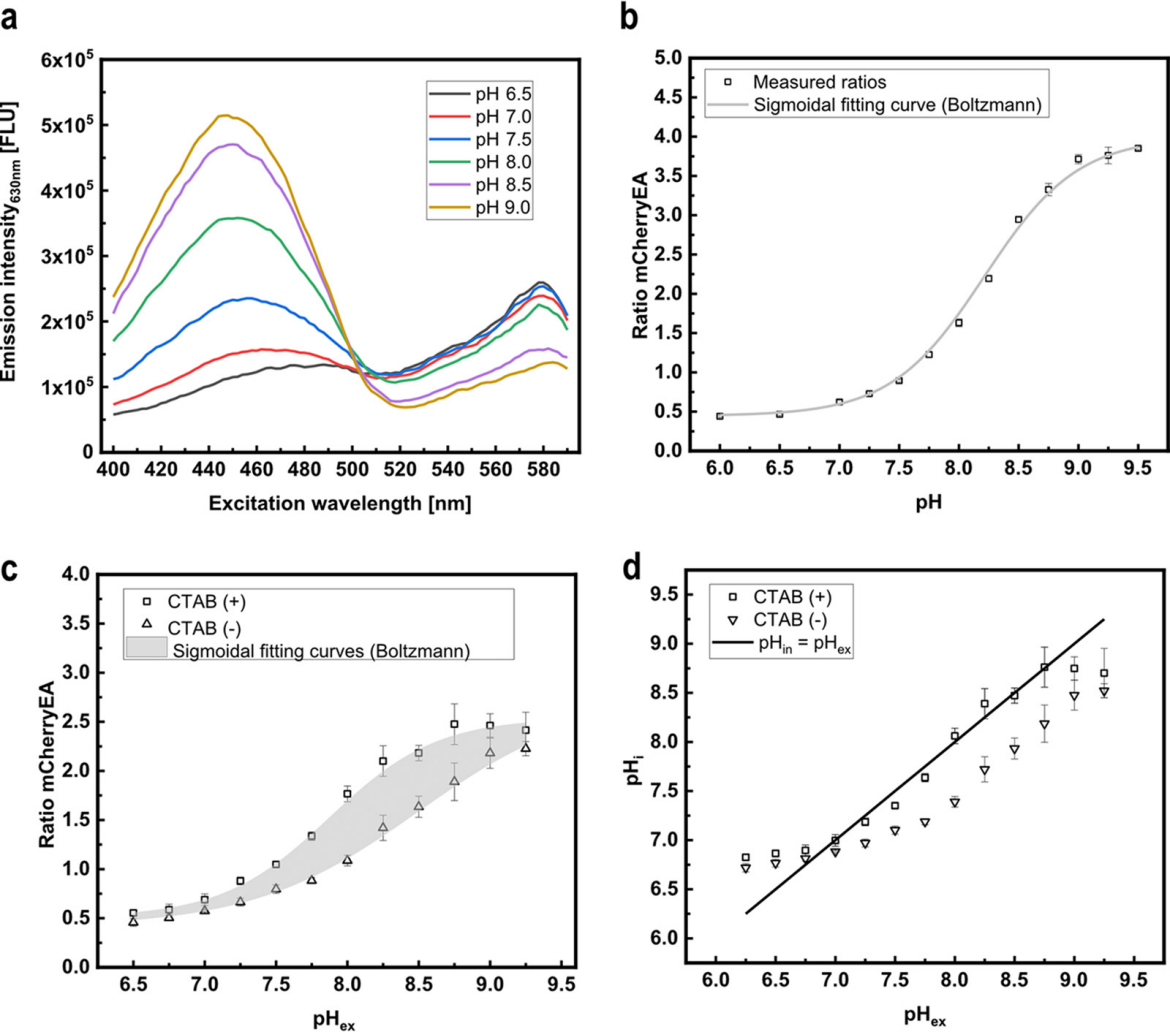
*In vitro* characterization of the pH biosensor mCherryEA using crude cell extracts of Escherichia coli DH5α (pEKEx2_*mCherryEA*). Spectral biosensor response upon changes in the respective pH (a) and the corresponding calculated pH-dependent ratios (b). mCherryEA biosensor ratio in E. coli DH5α (pEKEx2_*mCherryEA*) with and without the addition of cetyltrimethylammonium bromide (CTAB; 0.05% [wt/vol]) (c) and the calculated internal pH values of permeabilized cells compared to those of nonpermeabilized cells (d). The biosensor protein was produced in E. coli DH5α (pEKEx2_*mCherryEA*). Cell extracts were prepared in 1 M potassium phosphate buffer (PBS) buffer with different pH values. For *in situ* characterization, E. coli DH5α (pEKEx2_*mCherryEA*) was resuspended in 1 M PBS buffer with different pH values, and the cell suspensions were subsequently transferred to black 96-well plates. Fluorescence was measured before adding CTAB and after the addition of CTAB (incubation for 5 min in the dark prior to repeating the fluorescence measurements). The ratio of the biosensor mCherryEA was calculated by dividing the emission intensity (630 nm) obtained with an excitation at 454 nm by an excitation of 580 nm. Error bars represent standard deviation calculated from at least three replicates. Curve fitting was conducted using a sigmoidal fit (Boltzmann) in Origin. Fluorescence measurements were conducted in a SpectraMax iD3 plate reader.

The mCherry variant with I158E and Q160A amino acid exchanges, originally engineered to support excited-state proton transfer for generating a long Stokes shift variant, exhibits at neutral pH two excitation peaks, corresponding to the protonated and deprotonated chromophore, and a single emission peak (40). Based on this property, the mCherry variant, named mCherryEA, was found to function as a ratiometric pH sensor protein because the protonation state of Glu158 is sensitive to the pH of the surrounding solution, which results in pH-dependent protonation of the chromophore (35). To generate a sensor plasmid encoding mCherryEA, the gene was synthesized and cloned into the backbone of the expression plasmid pEKEx2, resulting in the plasmid pEKEx2_*mCherryEA*. Analysis of the fluorescence properties of the pH biosensor protein mCherryEA in cell-free extracts of E. coli DH5α (pEKEx2_*mCherryEA*) at different pH values shows an expected ratiometric and pH-dependent change of the emission intensity at 630 nm (Fig. 1a), as previously described (35). In detail, an increase in pH was accompanied by an increased emission intensity obtained for an excitation at 454 nm (maximum), whereas the emission intensity upon excitation at 580 nm decreased (Fig. 1a). Consistently, no pH-dependent changes of fluorescence were detected in cell-free extracts of the empty-vector control strain E. coli (pEKEx2) (see Fig. S1a in the supplemental material). Based on the changes in fluorescence emission for an excitation of both 454 nm and 580 nm, the pH-dependent ratiometric response of the biosensor mCherryEA was calculated (Fig. 1b). As depicted in Fig. 1b, an approximately 8-fold increase in the ratio occurred with increasing pH values from 6.5 to 9.0. Taking into consideration that internal pH values between 7.4 and 7.9 have been reported for E. coli (30, 31, 39), the mCherryEA biosensor properties seem well suited to assess changes toward both more alkaline and more acidic internal pH values. For testing the *in vivo* functionality of the mCherryEA biosensor, E. coli DH5α (pEKEx2_*mCherryEA*) cells were suspended in potassium phosphate buffer (PBS) buffer with different pH values, and the ratios of the fluorescence signals for the biosensor mCherryEA were subsequently determined for each of the cell suspensions in a microplate reader. As depicted in Fig. 1c, increased ratios were determined for E. coli DH5α (pEKEx2_*mCherryEA*) suspensions in high-pH PBS buffer and low ratios for suspensions at low pH. The ratiometric biosensor curve obtained from mCherryEA in cell-free extracts (Fig. 1b) differed from these determined for suspensions of intact cells (Fig. 1c). This observation indicates that pH homeostasis might proceed in the intact cells, causing the internal pH to be different from the external. The addition of the quaternary ammonium surfactant cetyltrimethylammonium bromide (CTAB) to cells permeabilizes the cell membrane and disrupts the proton gradient across the cytoplasmic membrane, allowing the internal pH to become identical to the external (4, 41). Indeed, the addition of CTAB to the suspensions of E. coli DH5α (pEKEx2_*mCherryEA*) with different pH values resulted in a shift in the ratiometric mCherryEA biosensor signals (Fig. 1c). When the internal pH (pH_i_) values for the CTAB-treated cell suspensions were calculated based on the obtained ratios (Fig. 1d), pH_i_ values within the expected dynamic range of the biosensor were obtained (Fig. 1d). The pH_i_ for nonpermeabilized cell suspensions of E. coli DH5α (pEKEx2_*mCherryEA*), however, was different from the external pH (pHex) (Fig. 1d), indicating that the cells probably performed pH homeostasis to some extent, even in the absence of an energy source.

10.1128/msystems.00219-22.2 FIG S1 (a) Spectral scan of empty vector control strain Escherichia coli DH5α (pEKEx2) conducted with cell extracts and different set pH values. Fluorescence intensities of E. coli MG1655 (pXMJ19) colonies on agar plates before (b) and after (c) their treatment with different set pH values (potassium phosphate buffer [PBS] buffer plus cetyltrimethylammonium bromide [CTAB]). Download FIG S1, TIF file, 1.2 MB. Copyright © 2022 Hartmann et al. 2022 Hartmann et al. https://creativecommons.org/licenses/by/4.0/ This content is distributed under the terms of the Creative Commons Attribution 4.0 International license.

**FIG 2:**
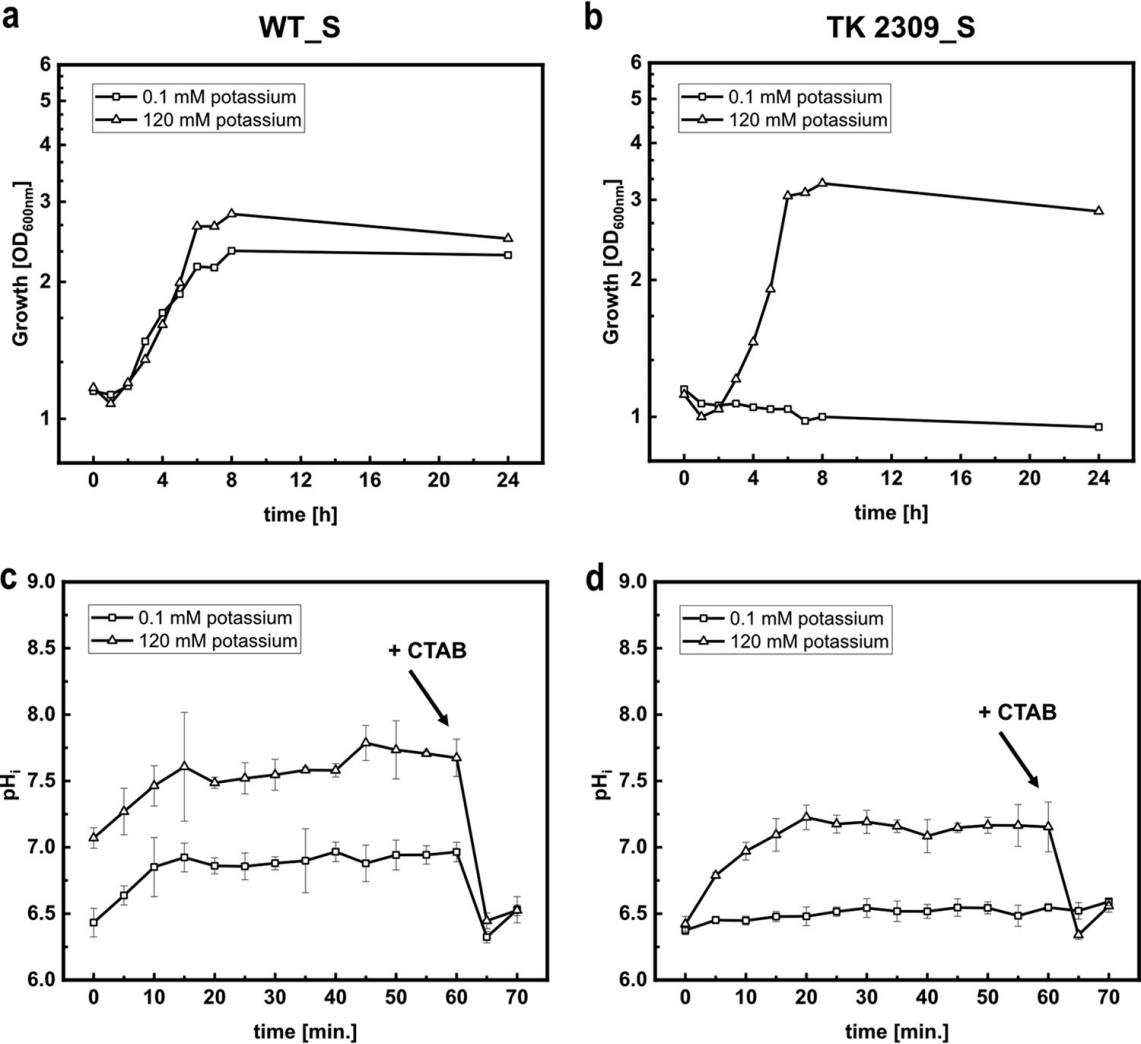
Growth (a, b) and on-line monitoring of the internal pH (pHi) (c, d) using the biosensor mCherryEA in minimal medium supplemented with 0.1 mM potassium (K_0.1_) and 120 mM potassium (K_120_) for E. coli MG1655 (pXMJ19_*mCherryEA*) (WT_S) (a, c) and E. coli TK2309 (pXMJ19_*mCherryEA*) (TK 2309_S) (b, d). Growth experiment was conducted in 50-mL cultures (500-mL shaking flasks, 37°C, 150 rpm) using K_0.1_ and K_120_ minimal media and 1% glucose (wt/vol). Growth was monitored via the optical density at 600 nm. For on-line monitoring, biosensor strains were prepared as described in Materials and Methods. Emission intensity at 630 nm was recorded upon excitation at 454 nm and 580 nm. At the end of the experiment, cetyltrimethylammonium bromide (CTAB; final concentration, 0.05% [wt/vol]) was added for sensor calibration purposes, as it allows equilibration of the internal and external environment. Error bars represent standard deviation from at least three replicates.

In order to test the pH biosensor mCherryEA for *in vivo* monitoring of internal pH values, we transformed E. coli MG1655, as well as the triple-mutant strain E. coli TK2309, which lacks all three major potassium uptake systems (Trk^−^, Kdp^−^, Kup^−^), with the sensor plasmid pXMJ19_*mCherryEA*, resulting in the sensor-equipped strains E. coli MG1655 (pXMJ19_*mCherryEA*) (WT_S) and E. coli TK2309 (pXMJ19_*mCherryEA*) (TK2309_S). For E. coli TK2309, a growth deficit was reported in the presence of less than 5 mM potassium in the growth medium (30). To verify this phenotype for the sensor-carrying strain TK2309_S, a growth experiment with WT_S and TK2309_S was conducted in minimal medium supplemented with 0.1 mM potassium (K_0.1_) and 120 mM potassium (K_120_) (Fig. 2a and b). Growth of the WT_S strain proceeded at rates of 0.12 h^−1^ and 0.2 h^−1^ at 0.1 mM and 120 mM potassium, respectively. (Fig. 2a). At low potassium concentrations, a growth deficit for TK2309 was observed (Fig. 2b) (30). However, this phenotype was abolished in the presence of 120 mM potassium, resulting in a growth rate of 0.22 h^−1^ (Fig. 2b).

For E. coli TK2309, a strong shift of pH_i_ toward an acidic value of 6.3 has been described upon incubation at an external pH of 6 and low potassium concentrations (30). This strong acidification in E. coli TK2309 does not occur in the presence of 120 mM potassium or in E. coli strains with at least one functional potassium uptake system (30). To reinvestigate the effects of potassium on pH_i_ by using the biosensor mCherryEA, we adapted a recently described real-time pH homeostasis experiment (11). For this purpose, WT_S and TK2309_S were precultivated for 24 h in LK medium (5 g/L yeast extract, 10 g/L Bacto tryptone, and 6.4 g/L KCl), followed by a cultivation step in K_30_ medium until the stationary phase was reached. Following that, the two strains were harvested, washed three times with 0.9% NaCl, and cultivated in K_0.1_ and K_120_ minimal media for 3 h. Finally, the cells were suspended in fresh K_0.1_ and K_120_ media (pH 6.0) containing 0.2% (wt/vol) glucose and then immediately transferred as 180-μL aliquots into 96-well-plates. Cells were incubated at 37°C, and fluorescence signals were recorded on-line for 60 min for determination of internal pH levels. As depicted in Fig. 2c, in the presence of 0.1 mM potassium, the internal pH of WT_S increased from an initial 6.5 to approximately 7.0 within 15 min of incubation. Upon addition of CTAB just before the experiment was ended, the sensor signal for pH_i_ dropped from 7.0 to approximately 6.5, which corresponds to the lower detection limit of the pH biosensor mCherryEA. A similar time course for pH_i_ was observed for WT_S in the presence of 120 mM potassium, for which the internal pH increased to approximately 7.7 after 15 min of incubation (Fig. 2c) before addition of CTAB caused the expected drop of the biosensor signal for pH_i_. The pH_i_ value of 7.7 measured for WT_S corresponds well to the pH_i_ values between 7.4 and 7.9 previously reported for E. coli wild-type (WT) strains incubated under similar conditions (11, 30, 39). Investigation of pH_i_ via the sensor mCherryEA in the potassium-uptake-deficient strain TK2309_S revealed that the sensor signal remained constantly at the lower detection limit of 6.5 for incubation in K_0.1_ medium with pH 6.0 (Fig. 2d). As expected for this external pH, addition of CTAB at the end of the experiment did not have any impact on the sensor signal for pH_i_. In the presence of 120 mM potassium, the initially recorded internal pH of 6.5 for TK2309_S increased within the first 20 min to around 7.3 ± 0.1 and remained stable at this level prior to CTAB addition at the end of the experiment, which caused the expected drop of the sensor signal (Fig. 2d). The pH_i_ values detected here for TK2309_S were below 6.5 in K_0.1_ medium and below 7.3 for K_120_ medium and correspond well with the internal pH values of 6.3 and 7.4 previously determined for this strain by the use of [7-^14^C]-benzoic acid (30).

Taken together, these experiments illustrate well the functionality of the sensor mCherryEA for the analysis of pH_i_ in liquid cultures of E. coli but also revealed restrictions of this method, which are set by the lower and upper pH detection limits of 6.5 and 8.75 of the sensor protein used.

### The biosensor protein mCherryEA can report the internal pH of E. coli colonies on agar plates.

**FIG 3:**
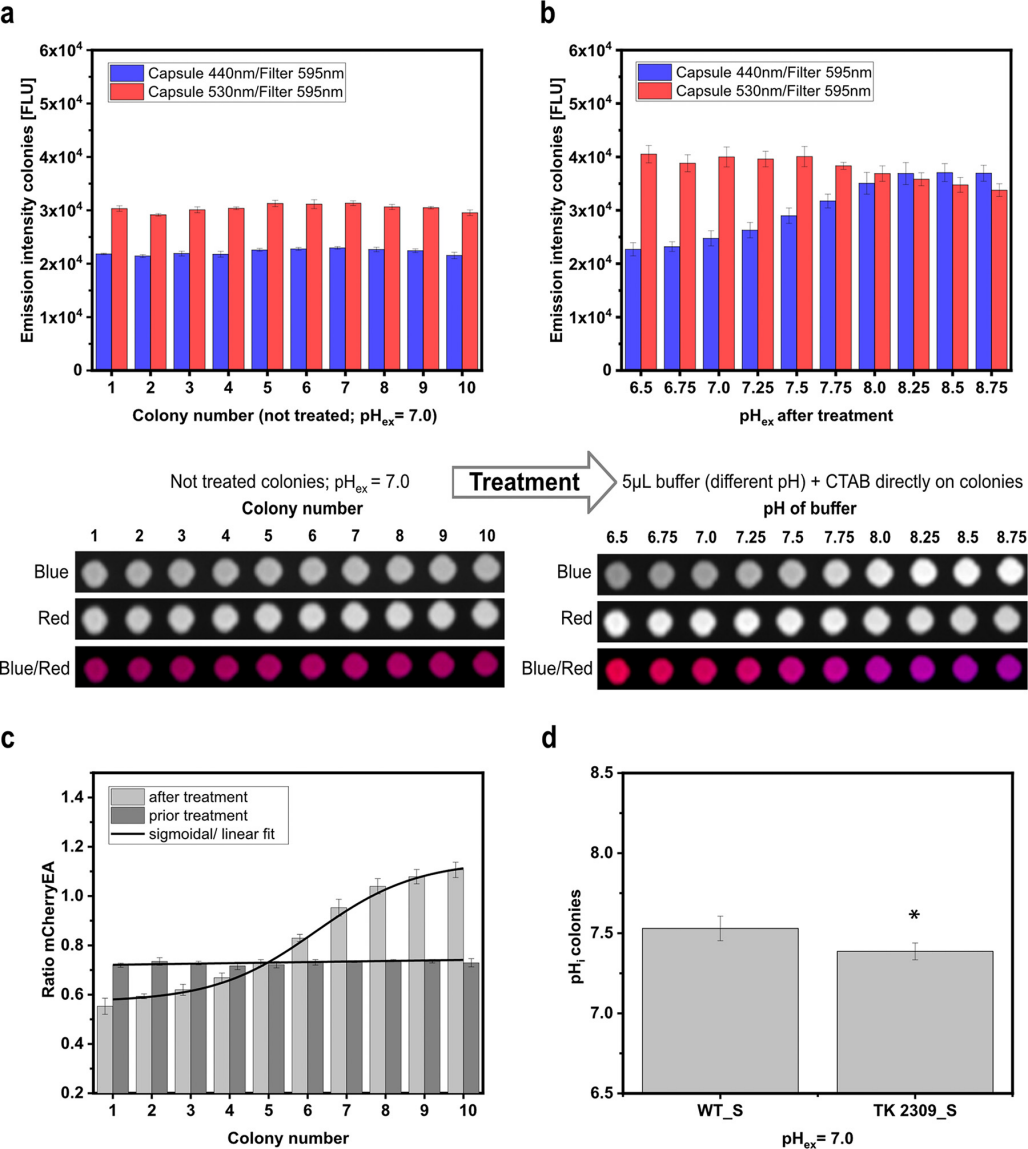
mCherryEA biosensor signals in E. coli MG1655 (pXMJ19_*mCherryEA*) arrayed colonies on agar plate without any treatment (a) and after adding buffer with different pH values directly to the respective colonies (b). Calculated ratios prior to and after treatment of the colonies (c) and determination of the internal pH values of E. coli MG1655 (pXMJ19_*mCherryEA*) (WT_S) and E. coli TK2309 (pXMJ19_*mCherryEA*) (TK 2309_S) (d). 1 M potassium-phosphate-buffer (PBS) buffer with different set pH values was supplemented with cetyltrimethylammonium bromide (CTAB; final concentration, 0.05% [wt/vol]). Internal pH values were analyzed with one-way analysis of variance (ANOVA) followed by Tukey’s test (not significant [n.s.], *P* > 0.05; *, *P* ≤ 0.05). Error bars represent standard deviation from at least three replicates.

To test if the sensor can be used to directly assess internal pH levels in colonies on agar plates, colonies of E. coli MG1655 (pXMJ19_*mCherryEA*) (WT_S) were spotted on rectangular plates containing screening-broth (SB) agar supplemented with 1 mM isopropyl-β-d-thiogalactopyranoside (IPTG). After the robot-assisted spotting, the agar plates were incubated for 20 h at 37°C, and the fluorescence of the colonies was then detected via imaging using an imaging system equipped for fluorescence analysis. The fluorescence detection was conducted using two different capsules for excitation (440 nm and 530 nm) and one filter (595 nm) to measure fluorescence emission. For colonies of WT_S, a mean fluorescence intensity of 2.22 × 10^4^ ± 552 fluorescence units (FLU) was measured for excitation at 440 nm, and a higher fluorescence intensity of 3.04 × 10^4^ ± 717 FLU when excited at 530 nm (Fig. 3a). For the empty-vector control strain E. coli (pXMJ19), colony fluorescence was more than four (0.48 × 10^4^ ± 90 FLU) and six (0.44 × 10^4^ ± 63 FLU) times lower upon excitation at 440 nm and 530 nm, respectively (Fig. S1b, c). These results show the proper expression of the biosensor protein mCherryEA in the WT_S colonies on SB agar plates supplemented with IPTG.

Genetically encoded FRPs have been shown to respond instantaneously to changes of the target internal parameter in liquid cultures, as shown in this study for the FRP mCherryEA in E. coli. This property should allow us to directly verify the biosensor functionality in colonies grown on agar plates. For this purpose, PBS buffer solutions with different set pH values and containing 0.05% CTAB were applied directly as 5-μL drops onto each of the colonies and then imaged again in the fluorescence imaging system. The changed fluorescence emission intensities after exposure of the colonies to the different buffer solutions revealed that the biosensor mCherryEA in the now-treated colonies responded in a ratiometric manner to the different pH values (Fig. 3b). In detail, the emission intensity at 595 nm for excitation at 440 nm increased when PBS buffer with higher pH values was added, and the emission intensity for the excitation at 530 nm decreased at lower pH values (compared to the initial intensities). By multiplexing the two fluorescence images derived for the treated colonies at different pH values, where excitation at 440 nm was assigned to the false color blue and excitation at 530 nm to the false color red, the effects of the exposure to low and high pH values could be visualized as a shift from red- to blue-colored colonies, respectively (Fig. 3b). The values for the fluorescence intensities obtained upon excitation at 440 nm and 530 nm determined for the colonies on the agar plate were used to calculate ratios for colonies before exposing them to the different set pH buffer solutions supplemented with CTAB and after their respective treatments. The ratio of the biosensor signal was shown to be in a narrow range (between 0.7 and 0.8) for all colonies before exposing them to the buffer solutions (Fig. 3c). Treatment of the colonies with the different pH buffer solutions containing CTAB resulted in a 2-fold ratio increase from 0.5/0.6 to 1.1/1.2 when buffer solutions with pH values of pH 6.5 or 8.75, respectively, were added (Fig. 3c). Based on the biosensor ratios for untreated colonies, an internal pH of 7.53 ± 0.08 was determined for E. coli MG1655 (pXMJ19_*mCherryEA*) (WT_S) (Fig. 3d). This is in accordance with an internal pH value of 7.43 ± 0.01 determined for liquid cultures of WT_S in SB medium (liquid) (Fig. S2b). In addition, the internal pH of E. coli TK2309 (pXMJ19_*mCherryEA*) (TK2309_S) grown as colonies on agar plates was determined to be 7.39 ± 0.05 (Fig. 3d). An internal pH of 7.22 ± 0.02 was measured for TK2309_S liquid cultures in SB medium (Fig. S2b). For liquid cultures, as well as the imaging method on agar plates, the internal pH of E. coli MG1655 was significantly higher than that of the TK2309 mutant. Despite the differences with respect to their internal pH, no growth deficit was observed in liquid SB medium for the TK2309_S strain compared to the WT_S strain (Fig. S2a). Taken together, the results successfully revealed the following:

(i) The sensor protein mCherryEA is functional in colonies on agar plates, and this method can be used to directly assess the pH_i_ from bacterial colonies on agar plates.
(ii) Imaging of the pH_i_ via the FRP (mCherryEA) signals from colonies on agar plates allows colonies with impaired pH homeostasis capabilities to be distinguished from those with a normal pH homeostasis, under conditions that do not negatively affect growth patterns.

10.1128/msystems.00219-22.3 FIG S2 (a) Growth experiment in screening broth medium (liquid) with the sensor strains E. coli MG1655 (pXMJ19_*mCherryEA*), as well as the potassium uptake-deficient triple mutant E. coli TK2309 (pXMJ19_*mCherryEA*). (b) Determination of internal pH values for both strains during the mid-exponential growth phase. Download FIG S2, TIF file, 0.1 MB. Copyright © 2022 Hartmann et al. 2022 Hartmann et al. https://creativecommons.org/licenses/by/4.0/ This content is distributed under the terms of the Creative Commons Attribution 4.0 International license.

### Fluorescent reporter protein-based screening of an E. coli transposon mutant library.

**FIG 4:**
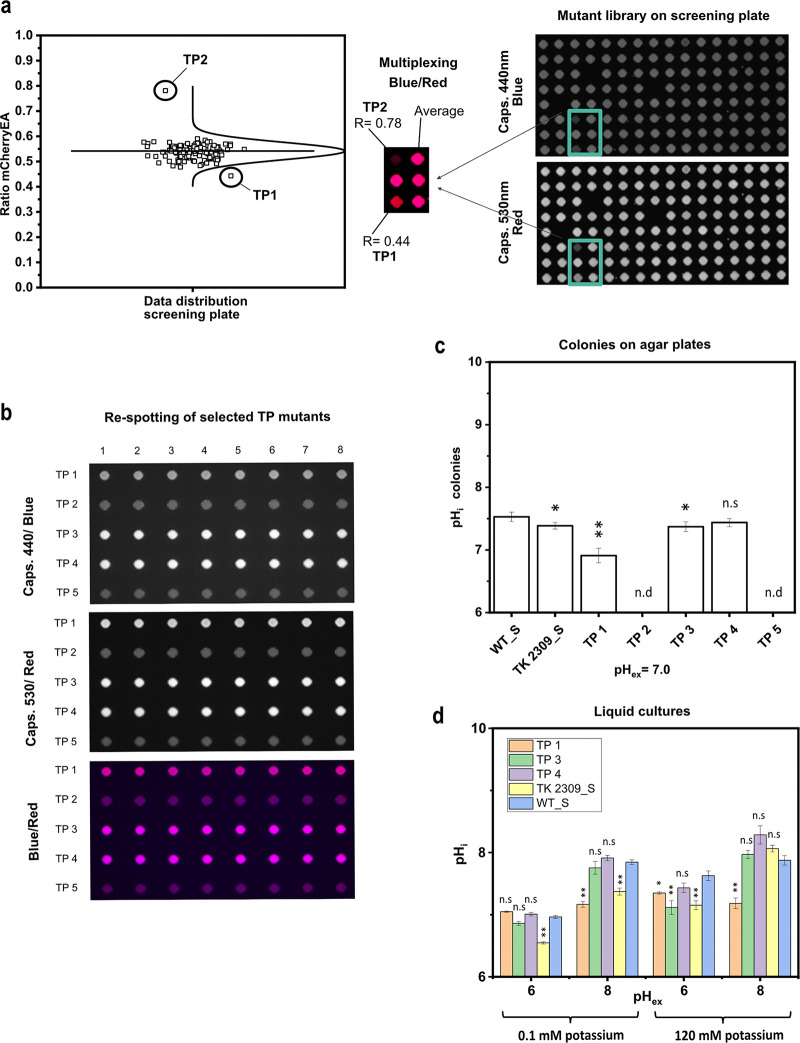
Fluorescence image of a screening plate with transposon-derived mutants of E. coli MG1655 equipped with the pH sensor plasmid pXMJ19_*mCherryEA* (a) and different selected transposon mutants respotted in eight replicates on SB agar plates (b). The respective internal pH values of eight replicates for the selected transposon mutants were determined and compared to E. coli MG1655 (pXMJ19_*mCherryEA*) (WT_S) and E. coli TK2309 (pXMJ19_*mCherryEA*) (TK2309_S) (c). Internal pH levels of selected transposon mutants were verified and compared to WT_S and TK2309_S strains in liquid media (minimal medium, K_0.1_ and K_120_) at different external set pH values (d). Error bars represent standard deviation of at least three replicates. Statistical analysis was performed via one-way ANOVA followed by Tukey’s test (n.s., *P* > 0.05; *, *P* ≤ 0.05; **, *P* ≤ 0.01). Internal pH was not determined (n.d.) for TP2 and TP5 mutants due to weak biosensor expression.

To finally validate the concept of using a FRP sensor to screen a strain library cultivated as colonies on agar plates, a Tn*5* transposon mutant library of E. coli MG1655 was created and transformed with the plasmid pXMJ19_*mCherryEA*. Linker PCR experiments revealed the expected heterogeneity of Tn*5* insertion sites for isolates of the mutant library before and after transformation of the sensor plasmid (Fig. S3a, b). Single colonies of the E. coli Tn*5* mutant library carrying pXMJ19_*mCherryEA* were picked randomly and transferred to single wells filled with SB medium in 96-well plates by the use of a QPix420 microbial colony picker. The 96-well microtiter plates were cultivated overnight and then colonies arrayed on SB agar plates (pH 7.0, 1 mM IPTG) using a RoToR HDA robot. After cultivation of the 384 clones arrayed on three SB agar plates (128 each) at 37°C for 24 h, the plates were imaged using the Vilber Fusion FX system. The average ratio of all colonies on the screening agar plates (Fig. 4a; see also Fig. S4a, b) was determined to be 0.50 ± 0.04. In Fig. 4a, the sensor ratio distribution and respective fluorescence image of one screening plate is depicted. Sensor analysis revealed a colony possessing a reduced ratio of 0.44 (TP1) and another colony with a drastically increased ratio of 0.78 (TP2). For all other colonies, such a rational decision was not possible. However, one further colony each located in the lower range of the colony ratios (TP3; 0.48) and in the higher (TP5; 0.59) were isolated for further analysis. Another interesting phenotype (Fig. S4a; screening plate 1) was isolated due to its different morphological structure compared to the other colonies growing on the screening agar plate, even though the ratio of this mutant was just slightly increased at 0.55 (Fig. S4a). From screening plate 2, no mutant was selected; however, the fluorescence images and analyzed ratios are provided in Fig. S4b.

10.1128/msystems.00219-22.4 FIG S3 Assessment of Tn*5* insertion sites diversity via linker PCR from randomly picked-up colonies from E. coli TOP10 Tn*5* mutant strain library (a) and again after introduction of the sensor plasmid pXMJ19_*mCherryEA* into the Tn*5* mutant strain library (b). Download FIG S3, TIF file, 0.4 MB. Copyright © 2022 Hartmann et al. 2022 Hartmann et al. https://creativecommons.org/licenses/by/4.0/ This content is distributed under the terms of the Creative Commons Attribution 4.0 International license.

10.1128/msystems.00219-22.5 FIG S4 Fluorescence images of screening plates 1 (a) and 2 (b) with all calculated mCherryEA biosensor signals derived from each colony of the respective screening plate. Download FIG S4, TIF file, 0.8 MB. Copyright © 2022 Hartmann et al. 2022 Hartmann et al. https://creativecommons.org/licenses/by/4.0/ This content is distributed under the terms of the Creative Commons Attribution 4.0 International license.

The follow-up fluorescence imaging analysis of these five transposon mutants on SB agar plates (pH 7.0) (Fig. 4b and c) revealed acidification of the internal pH for E. coli TP1 (pH_i_ of 6.91 ± 0.12) and E. coli TP3 (pH_i_ 7.37 ± 0.08) compared to the pH_i_ of 7.53 ± 0.08 measured for the reference strain E. coli MG1655 (pXMJ19_*mCherryEA*) (Fig. 4c). No significant difference of pH_i_ in comparison to that of the reference strain was measured for colonies of E. coli TP4 (pH_i_ 7.44 ± 0.06; Fig. 4c). In contrast to the sensor signals of E. coli mutants TP1, TP3, and TP4, the detected biosensor signals for the mutants TP2 and TP5 were very low (Fig. 4b). This observation indicates that the biosensor gene was weakly expressed in the two mutants E. coli TP2 (Tn insertions in *cusF*) and E. coli TP5 (Tn insertion in *ypaD*), which does not allow reliable analysis of the ratiometric signals of the pH sensor protein for these two strains (Fig. 4b and c).

For E. coli TP1, the Tn*5* insertion was mapped to the *rssB* gene (forward insertion at position +137, the nucleotide after which the transposon was inserted, starting from the first base of the start codon; Fig. S5a), encoding for the adaptor protein RssB, which is in the control of degradation of σ^S^ (RpoS) (42, 43). For E. coli TP3, the *trkH* gene for the major potassium uptake system TrkH (44) was found to be disrupted by the transposon (forward insertion at position +1303; Fig. S5b). For the Tn*5* mutagenesis-derived strain E. coli TP4, the gene *bcsA*, encoding the cellulose synthase BcsA (45), was found to be disrupted at position +658 (forward insertion; Fig. S5c). BcsA contributes to the synthesis of cellulose, a major structural component required for biofilm formation (45). Lack of BcsA could underlie the observed altered colony morphology of E. coli TP4 on the screening SB agar plate (Fig. S4a). After respotting dilution series of the selected Tn*5* mutant strains, however, the morphologically changed structure of the colony was not reproducible but was similar to the morphology of the other selected Tn*5* mutant strains (Fig. S6). For BcsA, no involvement in pH homeostasis has been reported. This is in agreement with the similar pH_i_ values measured for E. coli TP4 and the reference strain cultivated on SB agar plates (Fig. 4c).

10.1128/msystems.00219-22.6 FIG S5 Annotated genome of E. coli MG1665 and the respective polarity and position of the transposon insertion sites in *rssB* (a), *trkH* (b), and *bcsA* (c). Download FIG S5, TIF file, 0.3 MB. Copyright © 2022 Hartmann et al. 2022 Hartmann et al. https://creativecommons.org/licenses/by/4.0/ This content is distributed under the terms of the Creative Commons Attribution 4.0 International license.

10.1128/msystems.00219-22.7 FIG S6 Spotted dilution series on agar plates of selected E. coli TOP10 Tn*5* mutant strains. Download FIG S6, TIF file, 0.2 MB. Copyright © 2022 Hartmann et al. 2022 Hartmann et al. https://creativecommons.org/licenses/by/4.0/ This content is distributed under the terms of the Creative Commons Attribution 4.0 International license.

To further validate the results, pH_i_ of the Tn*5* mutants E. coli TP1, E. coli TP3, and E. coli TP4 was analyzed for liquid cultures in minimal medium at different pH_ex_ values and potassium concentrations after 1 h of incubation (Fig. 4d). In the presence of 120 mM potassium and a pH_ex_ of 6.0, pH_i_ values of 7.35 ± 0.02 and 7.15 ± 0.19 were measured for E. coli TP1 and E. coli TP3, respectively, which are significantly lower than the pH_i_ of 7.63 ± 0.08 measured for WT_S (Fig. 4d). In the case of E. coli TP3, this value is almost identical to the pH_i_ value of 7.14 ± 0.15 for TK2309_S (Fig. 4d). Upon exposure to a pH_ex_ of 8.0 and in the presence of 120 mM potassium, the pH_i_ values of both E. coli TP3 and the TK2309_S mutant (pH_i_ 7.97 ± 0.07 and pH_i_ 8.06 ± 0.05, respectively) were not significantly different from the pH_i_ of 7.88 ± 0.08 measured for WT_S, whereas the pH_i_ of E. coli TP1 remained at 7.18 ± 0.09 (Fig. 4d). At low potassium concentrations (0.1 mM) and a pH_ex_ of 6.0, neutral to slightly acidic pH_i_ values were determined for all strains. In detail, a pH_i_ of 7.05 ± 0.01 was measured for E. coli TP1 and a pH_i_ of 6.96 ± 0.03 for WT_S, and a pH_i_ of 6.86 ± 0.03 for E. coli TP3; the lowest pH_i_ of 6.55 ± 0.02 was measured in TK2309_S. Note that when setting an external pH of 8 and in the presence of low potassium concentrations, a still-neutral pH_i_ of 7.17 ± 0.1 was determined for E. coli TP1. In contrast, under these conditions, E. coli TP3 and WT_S revealed more alkaline pH_i_ values of 7.75 ± 0.1 and 7.85 ± 0.04, respectively. A pH_i_ of 7.37 ± 0.06 was determined for E. coli TK2309_S (Fig. 4d). As expected from the analyses on SB agar plates, for all four tested cultivation conditions, no significant differences in pH_i_ values of E. coli TP4 compared to those of the reference strain WT_S were observed (Fig. 4d).

These results show that the E. coli TP1 and E. coli TP3, identified via image analysis of colonies on SB agar plates as candidates with altered pH homeostasis properties, revealed Tn*5* insertions in genes known to be involved in pH homeostasis or pH stress adaptation (30, 46).

Surprisingly, for E. coli TP3, which carries the Tn*5* insertion within the *trkH* gene, the difference in pH_i_ (compared to that of the reference strain WT_S) was only detected to be significant in the presence of large amounts of potassium. However, the expression of genes for potassium systems is repressed in the presence of high potassium concentrations in E. coli (47, 48). This in turn can cause alterations of pH_i_ in *trkH*-deficient strains at potassium concentrations higher than 20 mM (30).

For the Tn*5* mutant defective in *rssB* (TP1), it should be highlighted that this strain maintained a stable neutral pH_i_ between 7.0 and 7.2, independent of the external conditions applied here with respect to both pH_ex_ and potassium concentrations. We hypothesized that this observed phenotype of E. coli TP1 is probably caused by constitutively high levels of RpoS, which in turn might lead to increased transcription of genes for the general stress response in E. coli and a stabilization of the internal pH (46, 49). To verify this hypothesis, we characterized the TP1 mutant strain with respect to its internal pH levels in real-time upon exposure to a broad range of different external pH values and quantified the protein levels of, e.g., RpoS and RssB via targeted proteomics.

### Elevated RpoS levels allow E. coli to maintain neutral internal pH levels over a broad range of external set pH values.

The observation that the transposon insertion in the *rssB* gene in the mutant E. coli TP1 selected here causes alterations of the internal pH prompted us to further characterize this mutant strain by performing a pH homeostasis assay. For this, the parental strain E. coli MG1655 (pXMJ19_*mCherryEA*) (WT_S) and the mutant strain E. coli TP1 were precultured as stated in Materials and Methods. Prior to performing the pH homeostasis assay, exponentially growing cells were harvested from shaker flasks (pH 7.0), resuspended in fresh minimal medium with different set pH values, and then immediately transferred to microtiter plates. Additionally, samples of these cultures grown at pH 7.0 were taken to be used for further analysis via targeted proteomics.

**FIG 5:**
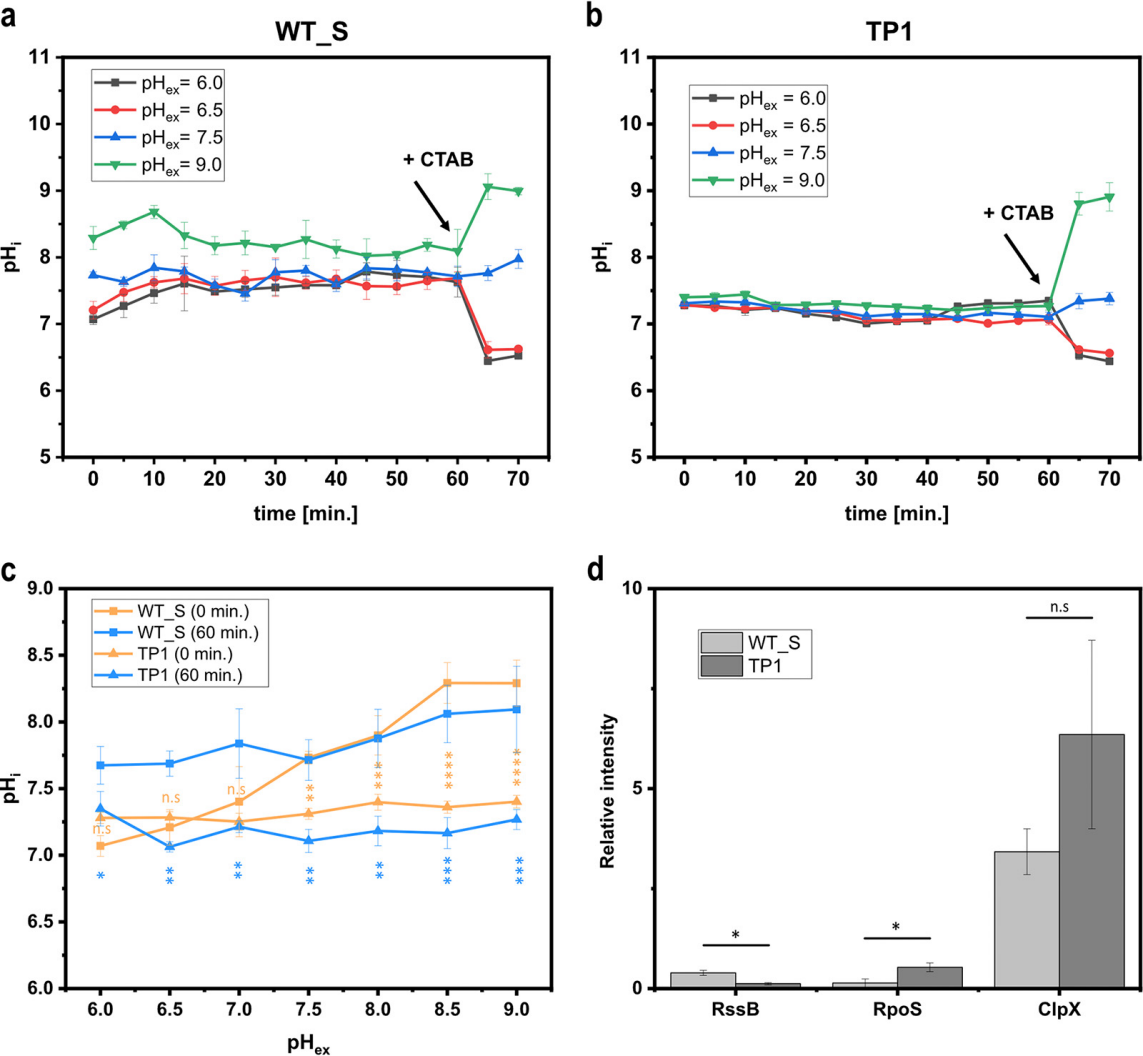
On-line monitoring of the internal pH using the biosensor mCherryEA in minimal medium for E. coli WT_S (a) and the selected Tn5*-rssB* mutant strain E. coli TP1, both equipped with the sensor plasmid pXMJ19_*mCherryEA* (b). Initial pH values recorded at the beginning of the pH homeostasis assay, as well as those after 60 min of incubation, are shown and compared for both background strains (c). Relative intensities of targeted proteins RssB, RpoS, and ClpX for both the E. coli WT_S and E. coli TP1 prior to conducting the pH homeostasis assay are shown (d). For on-line monitoring, biosensor strains were prepared as described in Materials and Methods. Emission intensity at 630 nm was recorded upon excitation at 454 nm and 580 nm. At the end of the experiment, cetyltrimethylammonium bromide (CTAB; final concentration, 0.05% [wt/vol]) was added for sensor calibration purposes, as it allows an equilibration of the internal and external environment. Error bars represent standard deviation of at least three replicates. Statistical analysis was performed via one-way ANOVA followed by Tukey’s test (n.s., *P* > 0.05; *, *P* ≤ 0.05; **, *P* ≤ 0.01; ***, *P* ≤ 0.001; ****, *P* ≤ 0.0001).

Internal pH monitoring in E. coli WT_S via the sensor protein mCherryEA revealed that initially, internal pH values were strongly affected by the external set pH (Fig. 5a and c). In detail, the internal pH shifted from 7.0 to between 8.5 and 8.7 upon increasing the external pH from 6.0 to 9.0 (Fig. 5a and c). During the course of incubation, pH levels were restored, and finally, after 60 min. of incubation, internal pH levels of 7.5 to 8.0 were measured for the reference strain E. coli WT_S (Fig. 5a and c). In contrast, the internal pH of the selected mutant strain E. coli TP1 was maintained in a narrow range between 7.1 and 7.2 from the beginning of the experiment, irrespective of the external set pH (Fig. 5b and c). Throughout the experiment, the internal pH levels were maintained stably in the *rssB* mutant E. coli TP1 (Fig. 5b), resulting in significantly lower internal pH levels for each externally adjusted pH value compared to those for the E. coli WT_S at the end of the experiment (60-min incubation) (Fig. 5c). Finally, the addition of CTAB induced the expected change of the biosensor signal due to the equilibration of external and internal pH, indicating the functionality of the biosensor protein mCherryEA under these conditions in both strain backgrounds (Fig. 5a and b).

These results confirm that the mutant strain E. coli TP1, in contrast to the reference strain E. coli WT_S, can stably maintain the internal pH in a neutral range upon exposure to a broad range of different external pH values. How can these differences in pH homeostasis be explained? The global stress sigma factor RpoS induces the expression of more than 500 genes involved in stress response and adaptation processes, such as those to acidic and alkaline pH stress (46, 50). Maintaining low RpoS levels during the exponential growth phase and under nonstress conditions is achieved by a combination of regulatory mechanisms, including low rates of synthesis and rapid degradation of RpoS via the ATP-dependent protease ClpXP (51–53). However, ClpXP does not recognize RpoS directly, as it needs to be delivered to the protease by the adaptor protein RssB in E. coli (54–56). Consequently, RpoS-stabilizing effects were shown to be present in *rssB* knockout strains of E. coli as a consequence of the absence of RssB-mediated delivery of RpoS to ClpXP (57). The selected mutant strain E. coli TP1 possesses a forward Tn*5* insertion in the *rssB* gene at position +137 (Fig. S7a). The insertion position belongs to the N-terminal domain of the RssB protein and is thus expected to disrupt the functionality of the gene, which in turn should lead to RpoS-stabilizing effects (Fig. S7a). To confirm this, RssB, RpoS, and ClpXP were quantified via targeted proteomics. The analysis revealed very low relative intensities (0.1 ± 0.03) for RssB in E. coli TP1 strain compared to those for the wild-type strain E. coli WT_S (0.4 ± 0.06) (Fig. 5d), indicating the absence of RssB in the mutant strain. In accordance with this, RpoS levels were significantly increased in the mutant strain compared to those in E. coli WT_S (5.3 times increased) (Fig. 5d). No significant difference was assessed for ClpXP, indicating that the elevated RpoS levels resulted from an impaired proteolytic degradation due to an impaired delivery of RpoS to the protease ClpXP by RssB (Fig. 5d).

10.1128/msystems.00219-22.8 FIG S7 (a) Amino acid sequence of the RssB protein with the N-terminal domain, the C-terminal domain, the identified position of the Tn*5* insertion, and the identified amino acid sequence via targeted proteomics. (b) Relative intensities of targeted proteins in E. coli MG1665 (pXMJ19_*mCherryEA*) and the selected E. coli TOP10 Tn*5* mutant strain carrying a transposon insertion in the *rssB* gene and the biosensor plasmid pXMJ19_*mCherryEA* (TP1). Download FIG S7, TIF file, 0.4 MB. Copyright © 2022 Hartmann et al. 2022 Hartmann et al. https://creativecommons.org/licenses/by/4.0/ This content is distributed under the terms of the Creative Commons Attribution 4.0 International license.

For alkaline pH homeostasis, organisms are equipped with powerful systems such as Na^+^/H^+^ antiporters, which are known to be involved in cell energetics and intracellular pH regulation (58). E. coli possesses two Na^+^/H^+^ antiporters, NhaA (59) and NhaB (60). Defective intracellular pH regulation was reported for E. coli mutants lacking the NhaB Na^+^/H^+^ antiporter (61). In agreement with this, a 200-fold increase of the NhaB protein level was measured in the *rssB*_S mutant E. coli TP1 compared to that in the reference strain E. coli WT_S (Fig. S7b). In contrast to the E. coli WT_S, this might have significantly contributed to the pronounced stabilization and acidification of the internal pH toward a neutral pH even when exposed to an external pH of 9.0 (Fig. 5b and c). Interestingly, to the best of our knowledge, RpoS-mediated induction of *nhaB* has not been reported thus far in the literature. In contrast, the induction of *nhaA*, encoding the second Na^+^/H^+^ antiporter NhaA in E. coli, can be initiated via two promoters (P1 and P2). Thus, P1 is involved in the Na^+^- and NhaR-dependent regulation, whereas P2 induction is dependent on RpoS in a Na^+^- and NhaR-independent fashion (58). Consequently, the accumulation of RpoS in the Tn*5*-*rssB* mutant E. coli TP1 is expected to cause higher NhaA levels; however, we were not able to target NhaA in either E. coli TP1 or E. coli WT_S.

Besides alkaline pH homeostasis, RpoS is also known to regulate the induction of proteins involved in acidic pH stress adaptation/homeostasis, such as the sophisticated acid resistance (AR) system AR1 and AR2 in E. coli MG1655 (28). The glutamate decarboxylase system (AR2) is known to be the most effective system to relieve acidic stress (28, 31, 62). The system is under multiple levels of control, including transcription factors for direct regulation of genes such as *gadA*, *gadB*, and *gadC*, encoding the two glutamate decarboxylase isoforms GadA and GadB as well as the antiporter GadC, respectively (63–66). The AraC-like transcriptional regulator GadX, produced from a gene immediately downstream of the *gadA* decarboxylase gene, has been shown to activate *gadA* and *gadBC* at any pH and to bind *in vitro* to the region around the Gad box (67). Moreover, expression of *gadX* proved to be dependent on the alternative sigma factor RpoS, making this circuit RpoS dependent (67, 68). Indeed, targeted proteomics revealed that GadX was significantly increased in the Tn*5*-*rssB* mutant strain compared to levels in the wild-type strain (9.8-fold increase) (Fig. S7b). As a consequence, the proteins GadB and GadC were found to be increased 15.4- and 13.9-fold, respectively, in the Tn*5*-*rssB* mutant strain E. coli TP1 compared to levels in the E. coli WT_S (Fig. S7b). The enzymes of the GAD system exhibit a pH optimum below 6.0 (62, 69). Thus, it is known to play a key role in counteracting strong acidic pH stress (pH < 2.5). Due to the properties of the pH sensor protein mCherryEA (dynamic range between 6.5 and 8.75), only mildly acidic conditions were tested in this study. To adequately capture low internal pH levels and their dynamics upon exposure to acidic stress conditions, pH biosensor proteins possessing lower pK_a_ values, such as pHluorin (4, 35) or the fluorescence resonance energy transfer (FRET)-based pH biosensor series named FluBpH (70), could be combined in future studies with the mCherryEA applied here.

Such a biosensor system could also be beneficial for screening to identify genes essential for coping with acidic stress, such as the amino acid decarboxylase systems in E. coli. In a recent study, a screening of 8,544 random E. coli transposon insertion mutants resulted in the identification of six genes essential for growth in lysogeny broth (LB) acidified to a pH of 4.5 (24). However, the authors stated that the threshold set for isolation of mutants with growth defect selected only the most sensitive mutants (24). A screen conducted under selective conditions using a sensor-based screening approach, as established here, could improve the screen sensitivity; however, adaptation to a biosensor set-up that also adequately captures acidic internal pH levels is therefore required.

### Conclusion.

High-throughput arrays of bacterial strain libraries on agar plates have become a versatile tool for phenotypic analysis aimed toward a comprehensive understanding of gene functions and interactions in microorganisms under various cultivation conditions (71–73). The use of genetically encoded sensors offers additional possibilities to investigate strain libraries for a single physiological parameter besides growth (1, 74, 75), and the combination of transcription factor-based biosensors (TFBs) with phenotypic arrays on agar plates has enabled comprehensive, nondestructive, temporally resolved gene expression studies (76). In contrast to this type of biosensor, fluorescent reporter proteins (FRPs) are commonly used in microorganisms for real-time monitoring of rapid internal kinetics in response to an environmental trigger (15, 17, 77). On the one hand, their fast response is a challenge for their use in FACS-based screening approaches. On the other hand, this characteristic enables measurement of the actual state of the cell. Here, the FRP-based genetically encoded pH sensor mCherryEA was successfully applied to screen, via fluorescence imaging, E. coli mutant colonies arrayed on agar plates. By exchanging the filters of the imaging system for those for the properties of other sensor proteins, the technology developed here can be applied to other metabolites and physiological states in colonies and thus become a versatile first step in comprehensive phenotypic analysis using FRP-based biosensors of genome-wide libraries of bacterial strains.

## MATERIALS AND METHODS

### Strains, media, and culture conditions.

Bacterial strains and plasmids used in this study are listed in Table 1. Cloning, as well as biosensor expression for the preparation of crude cell extracts, was carried out using E. coli DH5α cultivated in lysogeny broth (LB) medium (78). E. coli MG1655 and E. coli TK2309 were precultured in LK medium (5 g/L yeast extract, 10 g/L Bacto tryptone, and 6.4 g/L KCl). For main cultures, as well as short-duration cultivations to assess the impact of potassium on pH, K_0.1_, K_30_, and K_120_ media were prepared (79). For this purpose, K_0_ buffer [8.25 g/L Na_2_HPO_4_·2H_2_O, 2.8 g/L NaH_2_PO_4_·H_2_O, and 1 g/L (NH_4_)_2_SO_4_] was prepared, and a final buffer concentration of 0.1 mM KCl or 60 mM KCl was adjusted in order to get K_0.1_ and K_30_ media, respectively. K_120_ medium was prepared using K_120_ buffer instead [8 g/L K_2_HPO_4_, 3.1 g/L KH_2_PO_4_, and 1 g/L (NH_4_)_2_SO_4_]. All media and buffers were prepared using ultrapure water prepared using an Arium Pro ultrapure water purification system (Sartorius, Germany). Prior to inoculation, all K_x_ media were supplemented with 0.2% glucose, 0.4 mM MgSO_4_·7H_2_O, 0.6 μM (NH_4_)_2_SO_4_ × FeSO_4_·6H_2_O, and thiamine-HCl 0.0001% (wt/vol). For screening purposes, screening broth (SB) medium (5 g/L yeast extract, 10 g/L Bacto tryptone, 100 mM NaCl, and 50 mM KCl, buffered with 50 mM Tris; pH 7.0) was used. For preparation of agar plates, 16 g/L agar was added to each respective medium. Strains carrying plasmids and transposons were cultivated in the presence of kanamycin (50 μg/mL) or chloramphenicol (20 μg/mL). If required, 1 mM IPTG was added to induce expression of the gene for the biosensor. For fluorescence imaging of agarose plates, 50 mL of the medium was used for each plate (SBS-format PlusPlates; Singer Instruments, United Kingdom) and supplemented with black food dye (30 μL/plate) to reduce the fluorescence background from the medium.

**TABLE 1:** Bacterial strains and plasmids used in this study

Bacterial strain or plasmid	Description^*a*^	Reference or source
Escherichia coli strains		
E. coli DH5α	F^−^ φ80d*lacZ* Δ(*lacZYA*-*argF*) *U169 deoRsupE*44Δ*lacU169* (φ80*lacZ*ΔM15) *hsdR17 recA1 endA1* (r_K_^−^ m_K_^+^) *supE44gyrA96 thi-1 gyrA69 relA1*	81
E. coli DH5α (pEKEx2)	E. coli DH5α carrying the shuttle vector pEKEx2	This study
E. coli DH5α (pEKEx2_*mCherryEA*)	E. coli DH5α carrying a derivative of the shuttle vector pEKEx2 for IPTG-inducible expression of the *mCherryEA* gene	This study
E. coli TOP10	F^−^ *mcrA* Δ(*mrr-hsd*RMS-*mcrBC*) φ80*lacZ*ΔM15 Δ*lacX74 recA1 araD139* Δ(*ara-leu*)7697 *galU galK* λ^−^ *rpsL*(Str^r^) *endA1 nupG*	Invitrogen
E. coli MG1655	F^−^ λ^−^ *ilvG-rfb-50rpH-1*	82
E. coli MG1655 (pXMJ19)	E. coli MG1655 carrying the vector pXMJ19	This study
E. coli MG1655 (pXMJ19_*mCherryEA*)	E. coli MG1655 carrying a derivative of pXMJ19 for IPTG-inducible *mCherryEA* expression	This study
E. coli TK2309	F^−^ *thi rha lacZ nagA trkD1 trkA405 kdp*::Tn*10*	83
E. coli TK2309 (pXMJ19_*mCherryEA*)	E. coli TK2309 carrying a derivative of pXMJ19 for IPTG-inducible *mCherryEA* expression	This study
Plasmids		
pEKEx2	Expression vector; p*tac lacI*^q^ Km^r^	84
pXMJ19	Expression vector; p*tac lacI*^q^ Cam^r^	85
pEKEx2_*mCherryEA*	pEKEx2 derivative for IPTG-inducible *mCherryEA* gene expression	This study
pXMJ19_*mCherryEA*	pXMJ19 derivative for IPTG-inducible *mCherryEA* gene expression	This study

a Str, streptomycin; Km, kanamycin; Cam, chloramphenicol; ^r^, resistance.

### Genetics.

Gene fragment synthesis was carried out by Integrated DNA Technologies (IDT) (Denmark). The sequences are provided in Table S1 in the supplemental material. For amplification of the gene fragments, primer sets were designed using NEBuilder. All primers are listed in Table S1. Expression plasmids were assembled using the Gibson assembly kit (New England Biolabs, USA) according to the manufacturer’s instructions. After transformation, recombinant strains were selected using LB agar plates supplemented with appropriate antibiotics. Recombinant E. coli MG1655 and TK2309 strains were selected on LK medium agar plates with appropriate antibiotics. The plasmids were analyzed via PCR, restriction digests, and DNA sequencing (Eurofins Genomics, Germany).

10.1128/msystems.00219-22.1 TABLE S1 Nucleotide sequences of gene fragments and primers used in this study. Download Table S1, TIF file, 0.5 MB. Copyright © 2022 Hartmann et al. 2022 Hartmann et al. https://creativecommons.org/licenses/by/4.0/ This content is distributed under the terms of the Creative Commons Attribution 4.0 International license.

### Transposon mutagenesis library generation and introduction of pXMJ19-mCherryEA into the library.

The EZ-Tn*5* <KAN-2> Tnp transposome (Epicentre Biotechnologies, USA) was introduced into E. coli TOP10 cells by electroporation. Cells were subsequently spread-plated on LB agar plates containing 50 μg/mL kanamycin and incubated overnight at 37°C. Single colonies from the spread plates were analyzed to determine the diversity of insertion sites via linker PCR as described below. Pools of 5 × 10^3^ to 6 × 10^3^ colonies were collected and frozen at −80°C in 0.9% NaCl containing 30% glycerol. For the introduction of the sensor plasmid, 100 μL of these glycerol stocks was used to inoculate 2 mL LB containing 50 μg/mL kanamycin and then cultivated overnight on a shaker at 180 rpm and 37°C. Then, 2 mL of the preculture was used to inoculate 50 mL LB containing 50 μg/mL kanamycin in a 500-mL flask and cultivated at 200 rpm and 37°C until the culture reached an optical density at 600 nm (OD_600_) of 0.4; then, competent cells were prepared as described previously (80). The plasmid pXMJ19_*mCherryEA* was transformed into the mutant library competent cells using electroporation. Transformants were spread-plated on LB plates containing 50 μg/mL kanamycin and 20 μg/mL chloramphenicol and used for further screening.

### Identification of Tn*5* insertion sites using linker PCR.

Genomic DNA was extracted from 1.5-mL cultures using the GenElute bacterial genomic DNA kit (Merck, Germany). Linker PCR was used to test individual transformant colonies and to determine the diversity of insertion sites. Genomic DNA (2 μg) was digested with the AluI restriction enzyme (New England Biolabs, USA) and purified using an illustra GFX PCR DNA and gel band purification kit (GE Healthcare, USA). The linker was generated by annealing 100 μM each oligonucleotides P2-FW (Table S1) and P3-RV (Table S1) in an annealing buffer (10 mM Tris, 50 mM NaCl, and 1 mM EDTA; pH 8.0) after incubation at 95°C for 2 min, followed by cooling to 25°C for 1 h and chilling to 4°C. The linker was then ligated to the ends of restriction fragments (50 ng) using T4 DNA ligase (New England Biolabs, USA). The ligated DNA templates were cleaned by an illustra GFX PCR DNA and gel band purification kit (GE Healthcare, USA). Finally, linker PCR was carried out with the ligated DNA template and transposon-specific oligonucleotides P4-FW (Table S1) and P5-RV (Table S1) using Phusion X7 and thermocycling conditions of 98°C for 30 s, followed by 35 cycles of 98°C for 10 sec, 55°C for 30 s, and 72°C for 1 min, with a final extension step of 72°C for 10 min. The resulting PCR samples were run on 2% agarose gels at 100 V for 25 min. The PCR products were sequenced (Eurofins, Germany), and the respective sequence used for a BLAST search (CLC Workbench 8.0) against the genome of E. coli TOP10 to identify the insertion sites.

### Fluorescence analysis.

Fluorescence measurements of liquid cultures were conducted in black flat-bottomed 96-well microplates (Thermo Fisher Scientific, Germany) using a SpectraMax iD3 multimode plate reader (Molecular Devices LLC, USA). Excitation scans were recorded by setting the excitation wavelength between 410 nm and 588 nm and the emission wavelength at 630 nm. For ratiometric analysis of the biosensor signal, the emission maxima obtained upon excitation at 454 nm and 580 nm were taken, and the corresponding biosensor ratio was calculated by dividing the former emission intensity by the latter.

### Imaging of arrayed colonies.

Fluorescent imaging was carried out using the photo documentation system Fusion FX (Vilber Lourmat, France). The Fusion FX system was equipped with capsules for excitation at 440 nm and 530 nm with set exposure times of 40 ms and 1,560 ms, respectively. Fluorescence was measured using a 595-nm emission filter. Images were analyzed using the Fusion FX EVOLUTION-CAPT software (Vilber Lourmat). For this, fluorescence intensities at 595 nm obtained upon excitation at 440 nm and 530 nm were determined for each colony and divided by each other to calculate the ratiometric biosensor signal. With this imaging technology (resolution of 3,872 by 2,592 pixels; average radius of colony = 5 mm; distance from camera device to colony = 20 cm), heterogeneity and gradients within a colony cannot adequately be resolved. Consequently, the highest measured fluorescence intensity obtained from a colony was taken for further analysis.

### In vitro and in situ characterization of biosensor protein.

For *in vitro* characterization, biosensor strains and empty-vector controls were cultivated in shaker flasks (50 mL LB medium with respective antibiotics) until an OD_600_ of 1 was reached. Subsequently, 1 mM IPTG was added in order to induce expression of the gene from the biosensor, and cultivation was continued for 16 h at 37°C and 180 rpm. For preparation of crude cell extracts, cells were harvested by centrifugation (4,000 rpm for 10 min at 4°C), and washed twice in 1 M potassium phosphate buffer (PBS) with different pH values set by titrating 1 M K_2_HPO_4_ with 1 M KH_2_PO_4_. Finally, the washed cells were resuspended in 1 mL of the 1 M PBS with at the respective pH. Disruption of the cells was conducted using a benchtop homogenizer (FastPrep FP120; Savant, Germany) at 6,000 rpm, 4 times for 30 s each. Cell debris were removed via centrifugation (13,000 × *g* for 20 min at 4°C), and 200 μL of the supernatant was transferred to black flat-bottomed 96-well microplates (Thermo Fisher Scientific, Germany). For further fluorescence analysis, a SpectraMax iD3 microplate reader was used as stated above.

For *in situ* characterization of the pH biosensor mCherryEA, cells precultivated as described for the *in vitro* characterization were washed with 1 M PBS with different set pH values and finally resuspended in PBS at the respective pH to an OD_600_ of 3. Aliquots of the cell suspensions (190 μL) were then transferred to black flat-bottomed 96-well microplates. Subsequently, 10 μL CTAB (0.05% [wt/vol]) was added to the wells, and the plate was incubated for 5 min at room temperature in the dark for permeabilization of the cell membrane as previously described (4, 10). Subsequently, fluorescence measurements for biosensor analysis were performed in a microtiter plate reader as described above.

### Robotic colony picking and spotting.

Prior to picking colonies, wells of 96-well microtiter plates (Greiner Bio-One B.V., Netherlands) were filled with 150 μL liquid SB medium. Cells from single colonies on transformation plates were picked up using a colony-picking robot (QPix 420; Molecular Devices. LLC, USA) mounted with a bacterial 96-pin picking head and then transferred to the designated well in the 96-well plate. The QPix robot was programmed to create a copy of the target 96-well plate in a second prefilled 96-well plate in order to get one working plate and one back-up plate for long-term storage purposes. Following the picking procedure, the plates were incubated for 16 h in plate holders at 37°C and 280 rpm. Subsequently, 150 μL glycerol (50% [vol/vol]) was added to the cultures of the back-up plate, which was then immediately stored at −80°C. The working plate was used as a source plate for robotic spotting using a RoToR HDA benchtop robot (Singer Instruments, United Kingdom) on rectangular OmniTray plates (Singer Instruments) with solidified SB medium as the target plate. Four liquid source plates were finally combined on one target solid plate in a 96-well to 384 spots mode. The OmniTray plates were prepared using 50 mL of each respective medium supplemented with 1 mM IPTG for biosensor expression. Prior to liquid-to-solid spotting, pins were rotated five times in the source 96-well plate in order to generate a homogenous mixture of the cell suspension. Spotting was conducted by setting an overshoot of 1.5 mm and a pin pressure of 7% using long 96-well pins (Singer Instruments). To avoid reflection from the plastic edges of the OmniTray plates, as well as effects resulting from the outer barrier of arrayed colonies, the outermost lines and rows of spotted colonies on each plate were excluded from further analysis, resulting in 16 by 8 colonies on each target agar plate.

### *In vivo* assay to assess pH homeostasis by use of the plasmid-encoded sensor protein mCherryEA in E. coli liquid cultures.

Single colonies of E. coli strains carrying the plasmid pXMJ19_*mCherryEA* were used to inoculate 5 mL of LK medium, incubated overnight for 16 h, and then used to inoculate 50 mL of LK medium supplemented with 1 mM IPTG to induce expression of the gene for the pH biosensor protein mCherryEA. Stationary cells were harvested via centrifugation (3,500 rpm for 5 min at 4°C) and washed twice in 0.9% NaCl. Finally, cells were resuspended in K_30_, and the suspension was then used to inoculate 50 mL of K_30_ medium supplemented with 1 mM IPTG. The next day, cells were harvested via centrifugation (3,500 rpm for 5 min at 4°C) and washed three times with 0.9% NaCl. Subsequently, cells were grown for 3 h in 50 mL either K_0.1_ or K_120_ medium, supplemented with 1 mM IPTG. Finally, an OD_600_ of 3 was adjusted by resuspending the cells in fresh K_0.1_ or K_120_ medium (0.2% glucose [wt/vol]) with different pH values. Then, 180 μL of each cell suspension was transferred to black 96-well plates. Incubation and fluorescence measurements were carried out using the SpectraMax iD3 plate reader (incubation temperature of 37°C with continuous orbital shaking at medium intensity). Biosensor signals were then recorded in intervals of 5 min for 1 h. At the end of the experiment, CTAB (final concentration, 0.05% [vol/vol]) was added manually to each well in order to verify the biosensor functionality via equilibration of external and internal conditions. Moreover, this signal was used for recalibration of the pH biosensor signals at the end of every experiment.

For further characterization, the reference strain E. coli MG1655 (pXMJ19_*mCherryEA*) and the transposon mutant E. coli Tn*5*-*rssB* (pXMJ19_*mCherryEA*), both carrying the biosensor plasmid, were cultivated similarly to the above-described procedure and in the presence of 120 mM potassium. Finally, one part of the culture was used for further analysis (targeted proteomics). Cultures were prepared according to the above-described procedure, and the pH homeostasis assay was performed in microtiter plates with externally set pH values ranging from 6.0 to 9.0 (in steps of 0.5).

### Reverse phase liquid chromatography.

For targeted proteomics, 500 ng peptides were loaded onto a 15-cm by 75-μm PepMap rapid separation liquid chromatography (RSLC) column packed with 2-μm C_18_ beads (ES803A; Thermo Fisher) and using an EASY-nLC 1200 chromatography system with a 2-cm by 75-μm Acclaim PepMap100 trap column packed with 3-μm C_18_ beads. The peptides were eluted from the column using a mixture of solvent A (0.1% formic acid, catalog no. LS118-212, Fisher Scientific) and solvent B (80% acetonitrile and 0.1% formic acid, catalog no. 15431423; Fisher Scientific) at a rate of 250 nL/min. The chromatographic gradient was from 6% to 60% solvent B over 60 min (from 6% to 23% over 43 min, from 23% to 38% over 12 min, and from 38% to 60% over 5 min, followed by wash steps from 60% to 95% for 3 min and 95% solvent B for 7 min).

### Targeted mass spectrometry.

A Q Exactive mass spectrometer (Thermo Fisher Scientific) was operated in parallel reaction monitoring mode for 70 min at an MS1 resolution of 60,000 with the AGC target set to 3 × 10^6^, maximum injection time set to 20 ms, and a scan range of 400 to 1,200 *m/z* to select 36 targets for MS2 analysis. MS2 scans used a resolution of 60,000 with the AGC target set to 3 × 10^6^, maximum injection time set to 256 ms, and an isolation window of 0.7 *m/z* at a normalized collision energy of 28 with the target *m/z* values listed in Data Sets S1 and S2 in the supplemental material.

### Liquid chromatography-mass spectrometry analysis.

Data analysis of targeted proteomics was performed using Skyline. The search engine included all entries for E. coli from the UniProt Database (accessed 10 December 2017). Carbamidomethylation of cysteine residues (+57.021 Da) was set as a static modification, while oxidation (+15.995 Da) was a variable modification. Peptides were identified and quantified with at least three most-intense product ions. Area under the curve was used for quantification of the peptide intensity. The results were postprocessed by normalizing the respective intensities to obtained intensities for a housekeeping protein (6-phosphofructokinase) to finally obtain relative intensities for each targeted protein.

### Statistical analysis.

All experiments were carried out in triplicates, and mean values from three biological replicates were compared via one-way analysis of variance (ANOVA) followed by Tukey’s test. Differences with a *P* value of <0.05 were considered significant. All data were plotted and visualized using Origin software.

### Data availability.

All data generated and analyzed during this study are included in this article and its supplemental material. Raw data sets are available from the corresponding author on reasonable request.

10.1128/msystems.00219-22.9 DATA SET S1 Mass isolation list for targeted proteomics. Download Data Set S1, CSV file, 0.00 MB. Copyright © 2022 Hartmann et al. 2022 Hartmann et al. https://creativecommons.org/licenses/by/4.0/ This content is distributed under the terms of the Creative Commons Attribution 4.0 International license.

10.1128/msystems.00219-22.10 DATA SET S2 Peptide and protein targets for targeted proteomics. Download Data Set S2, CSV file, 0.00 MB. Copyright © 2022 Hartmann et al. 2022 Hartmann et al. https://creativecommons.org/licenses/by/4.0/ This content is distributed under the terms of the Creative Commons Attribution 4.0 International license.
