# Supplementary figures and images for: Visualizing the pH in Escherichia coli Colonies via the Sensor Protein mCherryEA Allows High-Throughput Screening of Mutant Libraries
Source: mSystems. 2022 Apr 18;7(3):e00219-22. doi: 10.1128/msystems.00219-22 (PMC9238402; doi:10.1128/msystems.00219-22)

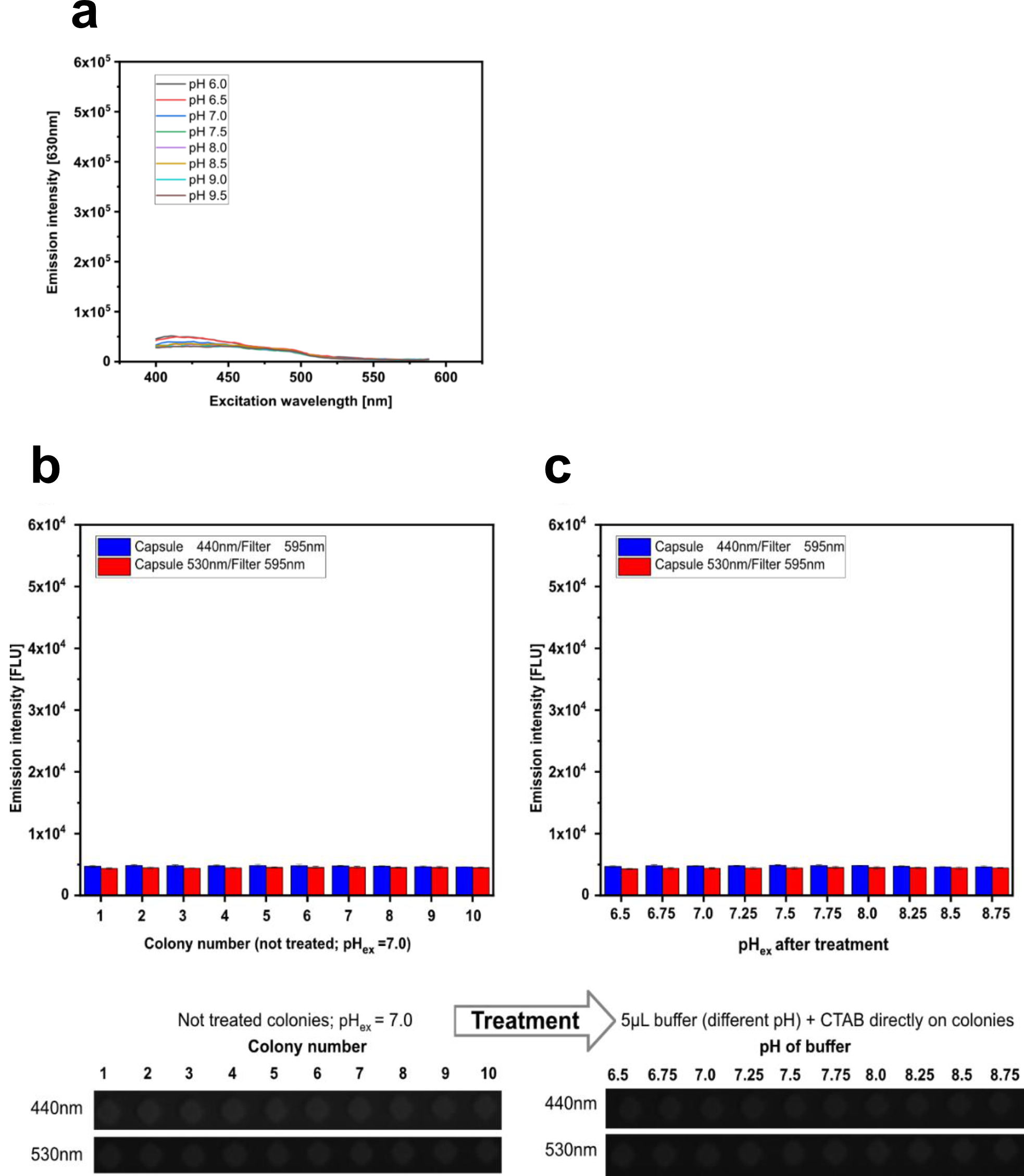

Supplement: FIG S1 [file msystems.00219-22-s0003.tif]

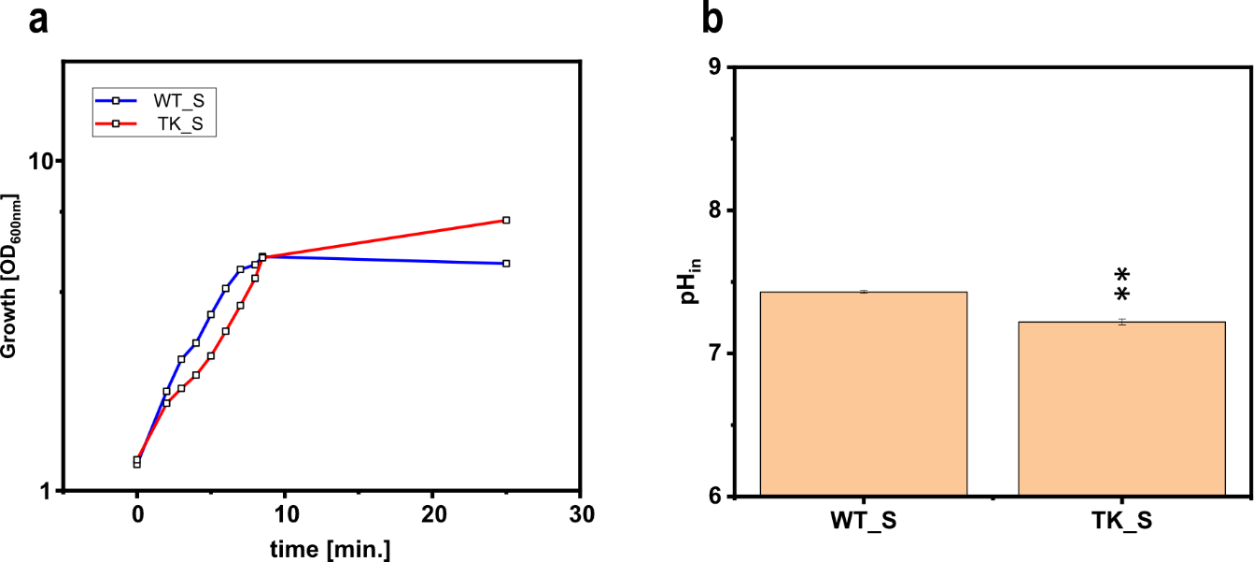

Supplement: FIG S2 [file msystems.00219-22-s0004.tif]

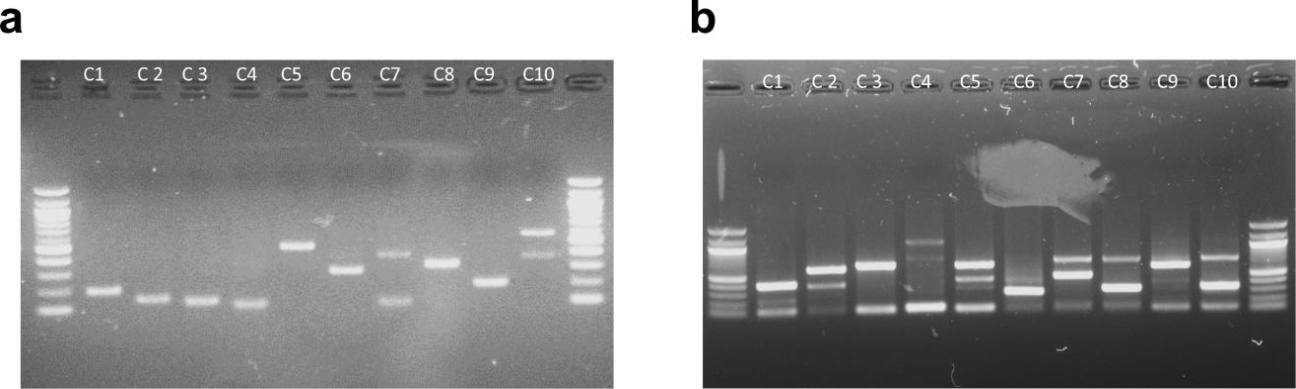

Supplement: FIG S3 [file msystems.00219-22-s0005.tif]

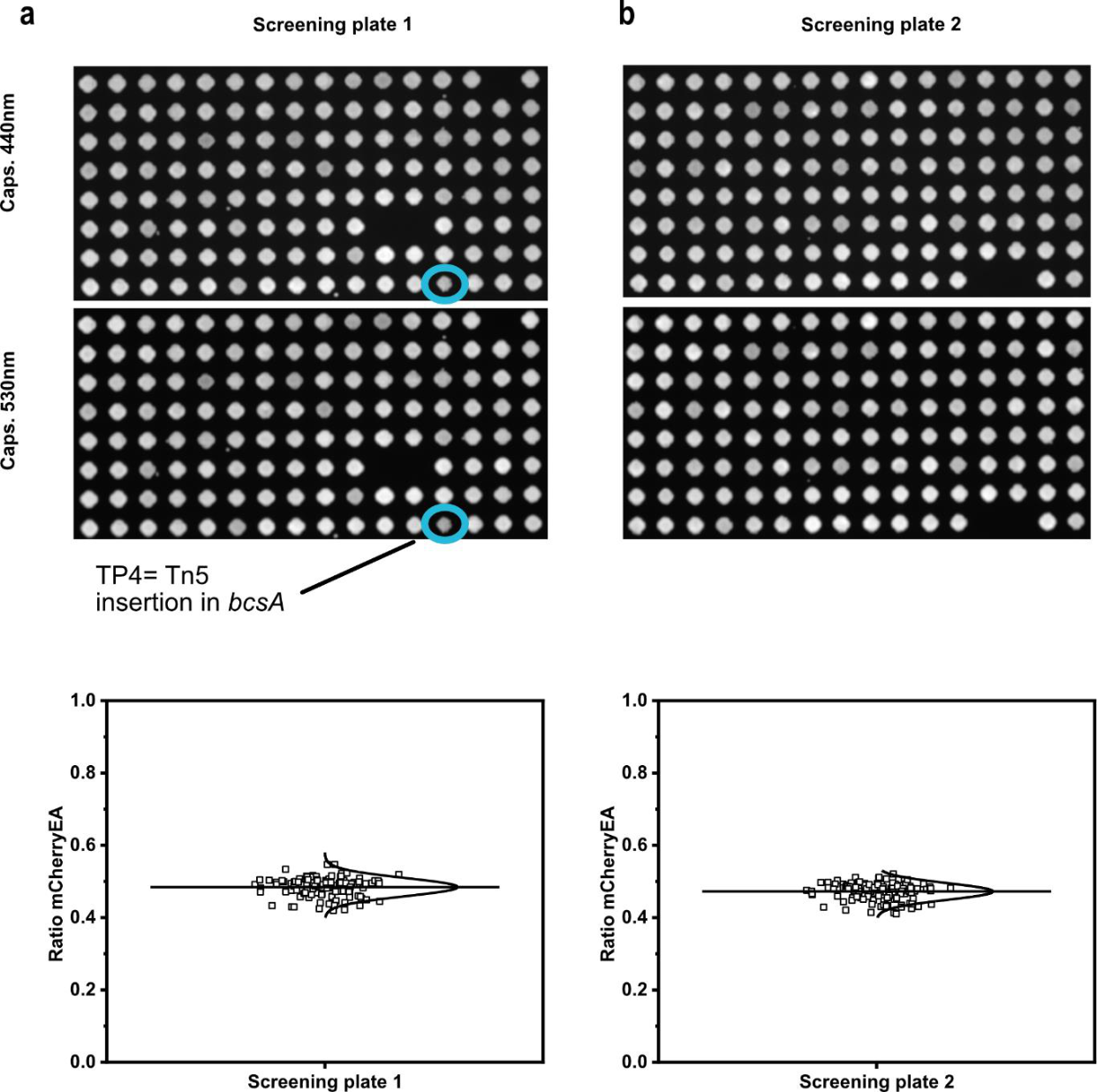

Supplement: FIG S4 [file msystems.00219-22-s0006.tif]

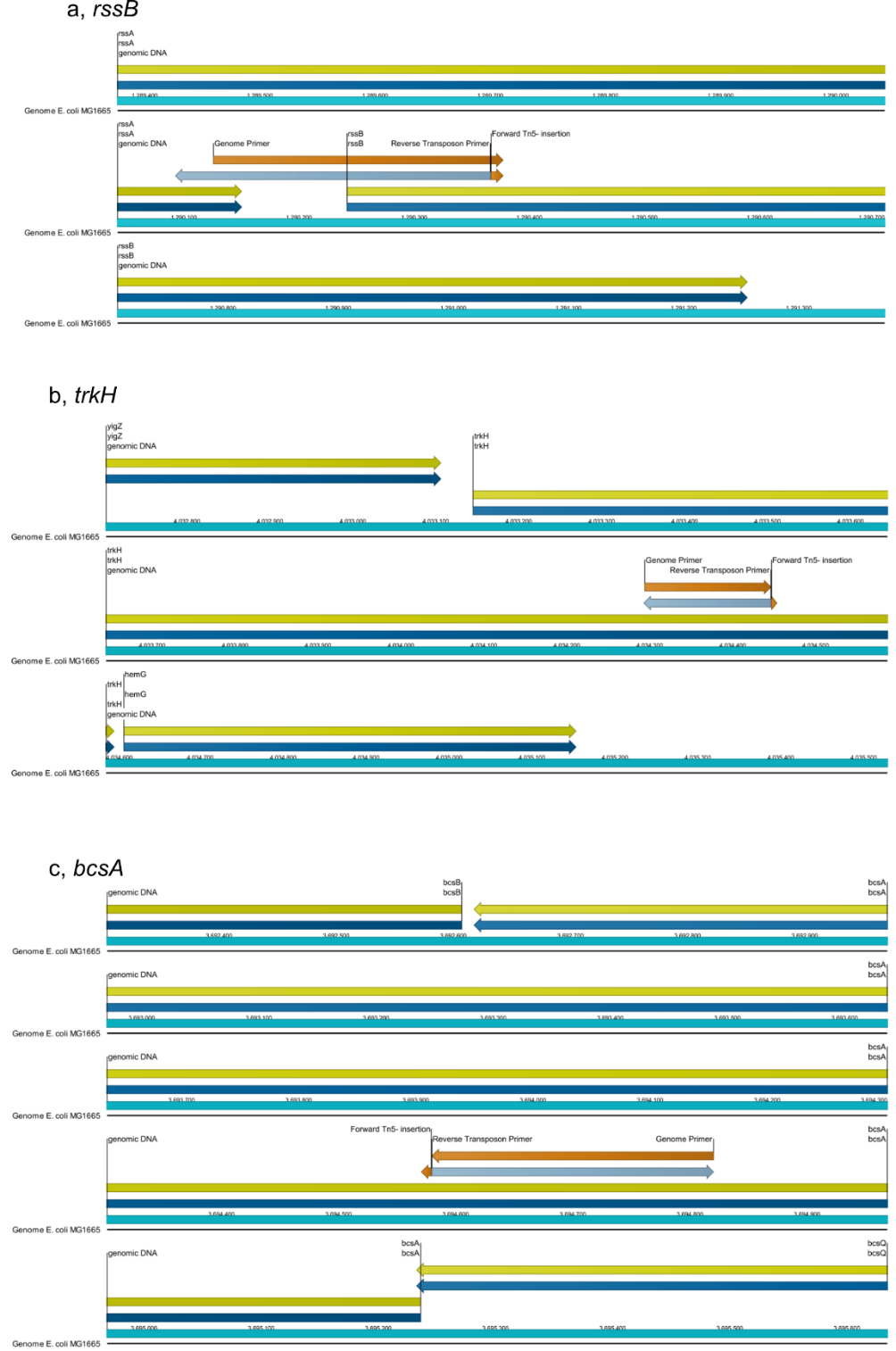

Supplement: FIG S5 [file msystems.00219-22-s0007.tif]

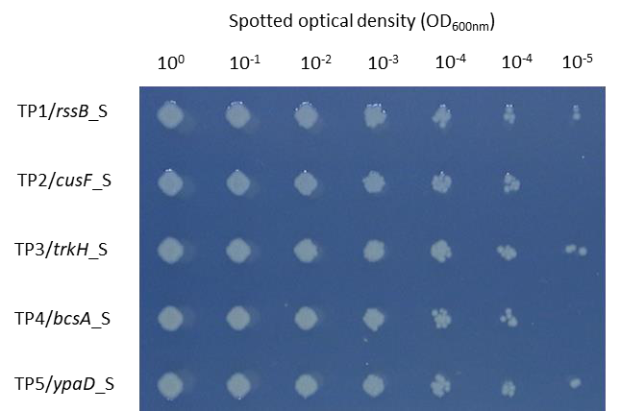

Supplement: FIG S6 [file msystems.00219-22-s0008.tif]

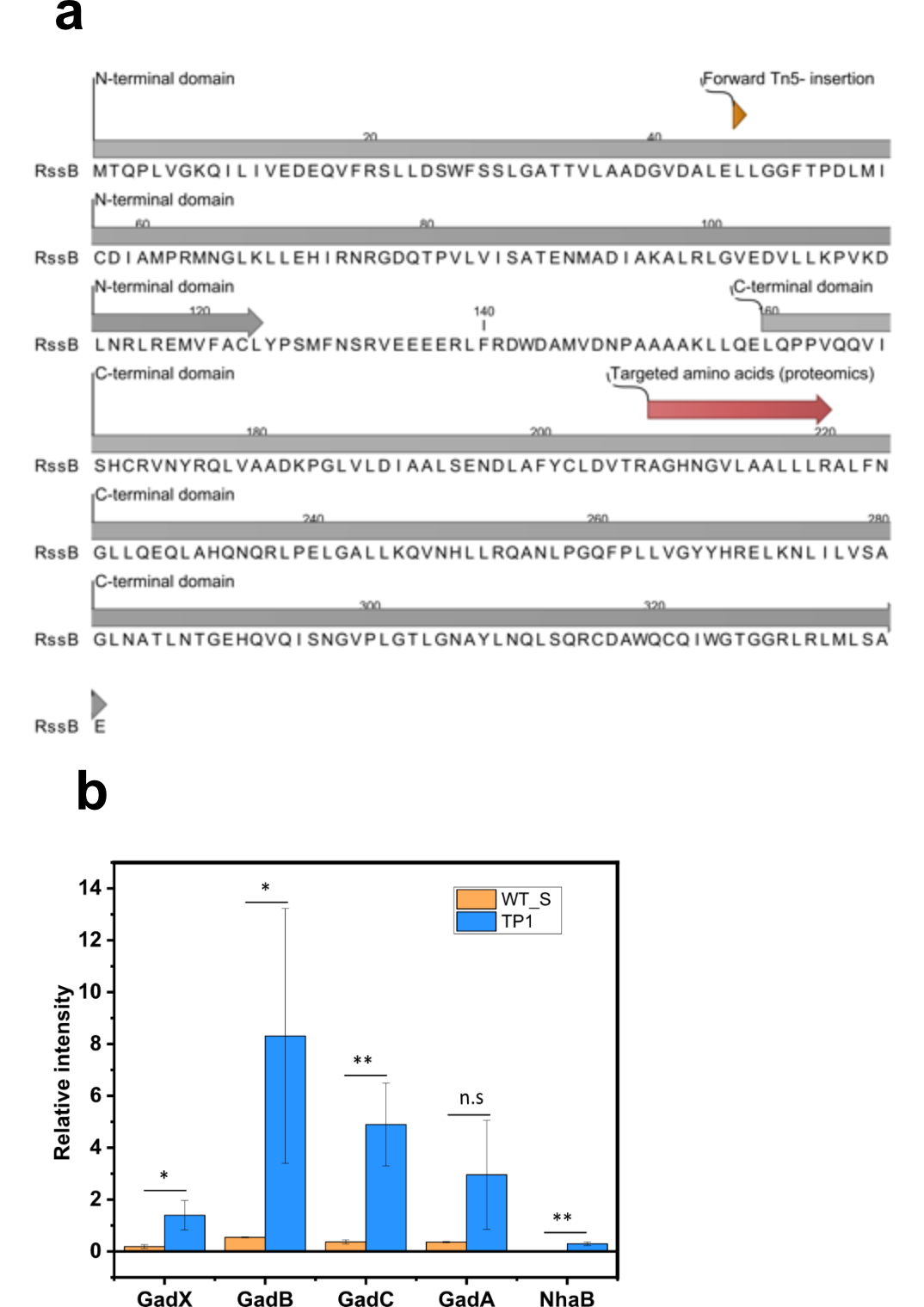

Supplement: FIG S7 [file msystems.00219-22-s0009.tif]

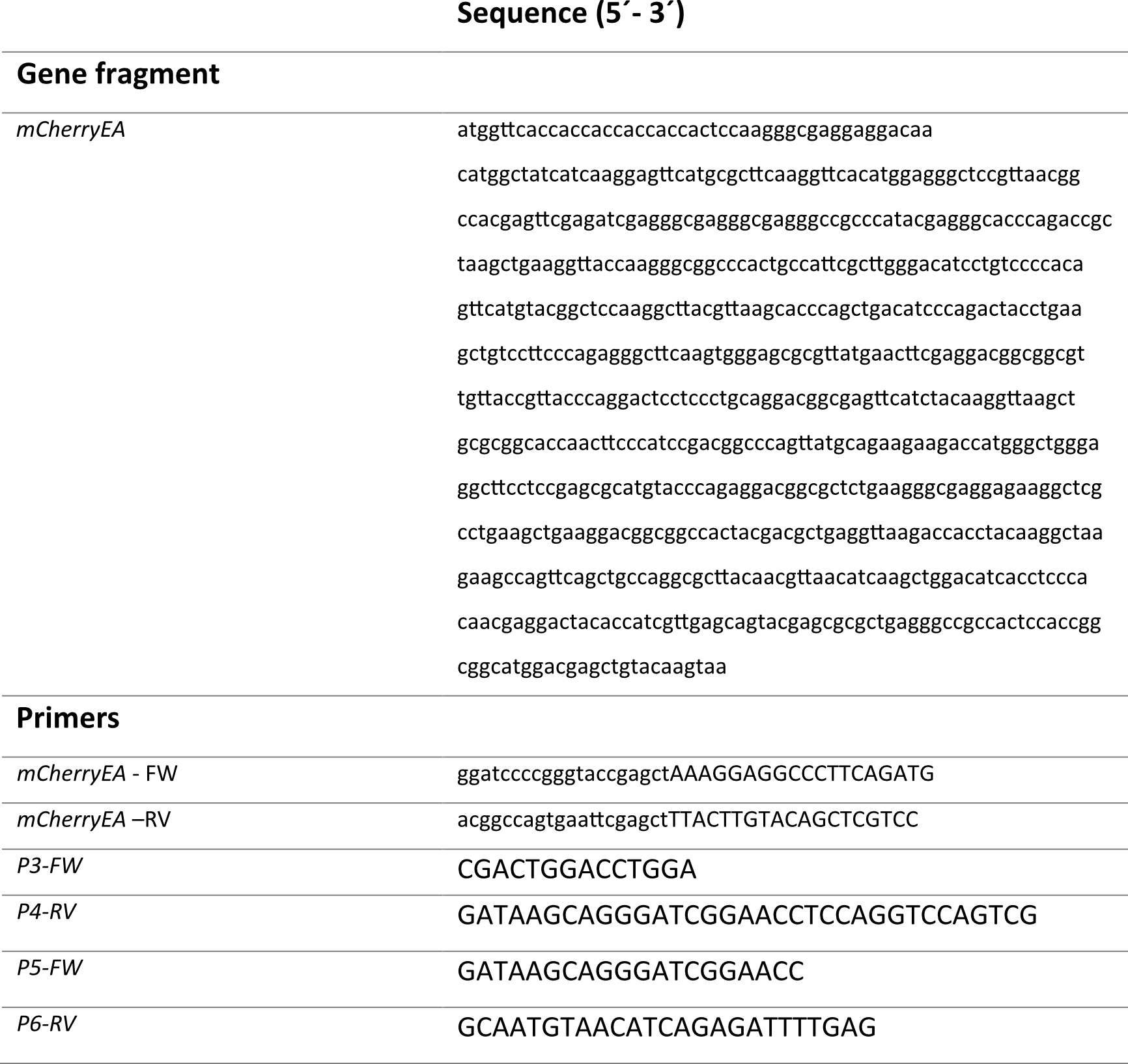

Supplement: TABLE S1 [file msystems.00219-22-s0010.tif]
